# Performance patterns and records in the world aquatics masters championships: Where do the most frequently represented nations among the top-ten masters swimmers come from?

**DOI:** 10.1371/journal.pone.0352386

**Published:** 2026-06-30

**Authors:** Sascha Moreitz, Wais Ahmad, Matthias Wilhelm, Aldo Seffrin, Marilia Santos Andrade, Pedro Forte, Arkadiusz Stanula, Lee Hill, Pantelis T. Nikolaidis, Katja Weiss, Thomas Rosemann, Beat Knechtle

**Affiliations:** 1 Centre for Rehabilitation & Sports Medicine, Inselspital Bern, University Hospital Bern, Bern, Switzerland; 2 Department of Radiology, Lucerne Cantonal Hospital, Lucerne, Switzerland; 3 Sanatorium Kilchberg, Kilchberg, Switzerland; 4 Postgraduate Program in Translational Medicine, Federal University of São Paolo, São Paolo, Brazil; 5 Department of Physiology, Federal University of São Paolo, São Paolo, Brazil; 6 Department of Sports Sciences, Instituto Politécnico de Bragança, Bragança, Portugal; 7 Department of Sports, Higher Institute of Educational Sciences of the Douro, Penafiel, Portugal; 8 Research Center for Active Living and Wellbeing (Livewell), Instituto Politécnico de Bragança, Bragança, Portugal; 9 Institute of Sport Sciences, Department of Swimming and Water Rescue, Academy of Physical Education in Katowice, Katowice, Poland; 10 Department of Pediatric Surgery, Research Institute – McGill University Health Centre, Montreal, Canada; 11 School of Health and Caring Sciences, University of West Attica, Athens, Greece; 12 Institute of Primary Care, University Hospital Zurich, Zurich, Switzerland; 13 Medbase St. Gallen Am Vadianplatz, St. Gallen, Switzerland; Universidade do Porto, PORTUGAL

## Abstract

**Background:**

Masters Swimming is a rapidly expanding sector of competitive aquatic sport and provides structured opportunities for adults to maintain long-term engagement in high-level swimming. Despite extensive global participation, limited evidence exists regarding the national origins of the most frequent Masters swimmers among the top-ten performers across all strokes, distances, and age groups. Previous research has focused primarily on isolated disciplines or open-water events. This study aimed to investigate participation and performance patterns of Masters swimmers competing at the World Masters and World Aquatics Championships from 1986 to 2024, with a specific focus on national patterns among top performers.

**Methods:**

A total of 204,005 long-course (50 m) swimming performance entries (94,312 women and 109,693 men) from 1986 to 2024 were extracted from the official World Aquatics archive and analyzed. The unit of analysis was the annual top-ten entries for each stroke, distance, sex, and age group, with each result treated as an independent performance record. Statistical differences between nationalities were assessed using Kruskal-Wallis tests with Bonferroni post-hoc adjustments. Descriptive data were presented using mean, standard deviation, and confidence intervals. Success was operationally defined as the frequency of a nation’s appearances in the annual top-ten fastest times for each stroke, distance, and sex. For descriptive purposes, nationalities were grouped into six categories: the top-five nationalities with the most appearances in the top-ten fastest times regarding the different swimming strokes and sex separately by distances and for each competition year, as well as one group of all other nationalities.

**Results:**

Among women, German swimmers were the most numerous in the top-ten for breaststroke, butterfly, and 50 m backstroke. Among men, however, U.S. swimmers were the most frequently represented across almost all distances and strokes, except for the successes of Brazilian athletes in the 50 m backstroke and Russian swimmers in the 50 m breaststroke. Across all years and disciplines, the most recurrent top-ten Masters swimmers came from the USA, Germany, Great Britain, Russia, Italy, and Brazil (“Big Six”). Performance differences between nationalities were significant across multiple events, with notable strengths among Russian swimmers in breaststroke and freestyle sprint events.

**Conclusions:**

The United States demonstrated the broadest and most consistent numerical superiority in Masters swimming performance over the 38-year period, by fielding the most frequent athletes among the top-ten performers, particularly among men. German women displayed exceptional success in breaststroke and butterfly, accounting for the majority of the athletes in the top-ten. These findings highlight persistent geographic patterns in Masters swimming excellence and may guide future research on environmental, sociocultural, training, and demographic factors contributing to national performance patterns.

## Introduction

Swimming is a widely practiced recreational and competitive sport that offers structured opportunities for physical activity, skill development, and lifelong participation [1]. Masters Swimming, in particular, emphasizes inclusivity: athletes compete in age-group categories, access organized training groups, and participate in both national and international competitions. Although some Masters athletes have prior elite experience, the Masters system is not intended to mirror professional swimming. Instead, it promotes long-term engagement, physical fitness, and community involvement [2]. Reflecting this, Masters Swimming has experienced sustained global growth over the past several decades [3].

Masters Swimming was first included in the FINA World Championships in 1984 (Following the establishment of the first national association, the International Federation of Amateur Swimming – known as FINA/Fédération Internationale de Natation Amateur – was founded in 1909 in London (https://britannica.com). It was officially renamed World Aquatics in 2023 under the slogan of a new vision and mission: a world united by water for health, life, and sport to ensure the ability to participate, compete in, and benefit from (https://www.worldaquatics.com). As the international governing body, World Aquatics is responsible for overseeing and regulating aquatic sports, including Masters Swimming, and organizing the prestigious international World Aquatics Championships (https://www.worldaquatics.com), formerly FINA World Championships.), with the inaugural Masters World Championships held in Tokyo in 1986 [4–6]. Since then, participation has expanded dramatically. The integration of the Masters Championships immediately following the elite FINA World Championships since 2015 further increased visibility and accessibility [4]. Annual increases in event participation reflect the continuing rise in Masters swimmer involvement worldwide.

Previous research has demonstrated that athletes from certain countries dominate particular sports. For example, Jamaica produces world-class sprinters [7], while East African nations, especially Kenya and Ethiopia, excel at middle- and long-distance running [8–10]. Geographic, genetic, socioeconomic, and environmental factors may influence these national performance patterns [11–19].

In swimming, however, literature examining demographic and national origin-based performance trends—particularly in Masters swimming—is limited. Existing research has focused primarily on open-water events [20,21–23] or single-stroke Masters disciplines such as butterfly [24], backstroke [25], and recently on freestyle [26]. No comprehensive study has evaluated all swimming strokes, distances, and age groups in Masters competition over multiple decades.

This study addresses that gap by analyzing national origins of the most frequent Masters swimmers among the top-ten performers at the World Masters and World Aquatics Championships from 1986 to 2024 across all strokes and distances. Given the long-established dominance of U.S. and Australian athletes in elite swimming [27,28], and the participation of former elite swimmers in Masters divisions, we hypothesized that U.S. athletes would be disproportionately represented among the the top-ten performers.

## Materials and methods

### Study design

This study used a retrospective observational design to examine participation and performance patterns among Masters swimmers competing in World Masters and World Aquatics Championships from 1986 to 2024. All data were publicly available through the official World Aquatics (formerly FINA) results archive and consisted exclusively of competition results from long-course (50 m) pool events. No interventions, human contact, or recruitment occurred.

### Data set and data preparation

The race data were obtained by Ahmad Wais and Sascha Moreitz from the official website of World Aquatics (www.worldaquatics.com). We sourced complete data for all World Masters and World Aquatics Championships held between 1986 and 2024, twenty in total (From 1986 until 2014, the FINA World Masters Championships were held as an independent event every two years. However, starting in 2015, the Masters category was integrated into the World Aquatics Championships S1 Table.), by downloading competition results directly from the official World Aquatics archive on their respective webpage (www.worldaquatics.com/masters/archives/masters-archives). The data set was then stored and organized using Excel spreadsheets. Corresponding to the date of the most recent published results, the data set we analyzed was last supplemented and updated on 3 March 2024.

From each swimmer, the year of competition, first name, last name, age, age group, swimming stroke, and distance were recorded. World Aquatics follows rigorous verification and publication procedures to ensure the accuracy and integrity of the information. This data is publicly accessible and can be validated and checked by any interested party. Additionally, we conducted internal cross-referencing and manual reviews to further ensure data integrity. Our dataset, as submitted to the analysis procedures, is available upon request to the leading author.

All competition data analyzed were from events held in long course meter (LCM) 50-meters pools. No competition occurred during the pandemic from 2020 to 2022; therefore, no data is available. This three-year gap reduces the total number of championship editions but does not introduce a systematic directional bias, as the analysis relies on cumulative frequency counts. It should be noted that athletes from Russia were not allowed to compete under their flag in 2023 and 2024 for political reasons. Their nationality was abbreviated to “NIA”. However, as they are included in the statistics from 1986 to 2019, their nationality was changed from “NIA” to “RUS”. This recoding restores continuity with the preceding 34 years of consistent national coding and affects only 2 of the 20 championship editions analyzed (To bound the potential impact of the NIA to RUS recoding on national rankings, we note the following. First, the NIA was applied to only 2 of the 20 championship editions analyzed (10%). Second, the primary outcome is the cumulative frequency of top-ten appearances across 1986–2024, aggregated over 18 additional editions in which Russian athletes competed under the RUS code. For any given event, the maximum possible contribution of the 2023–2024 editions to a national ranking is therefore bounded by the number of Russian top-ten entries across those 2 editions, added to a 34-year base of pre-existing RUS classifications. As a consequence, the recoding is ranking-preserving by construction: it restores historical coding continuity rather than altering the relative standing of nations whose representation is stable across 3 prior decades of data. Athletes from Russia were consistently among the top-nations in the events in which they now appear within the top-five, establishing that the recoding reinforces a longstanding representation rather than creating a novel one.).

During that period, a total of 204,005 swimmers (94,312 women and 109,693 men) competed in 50 m, 100 m, and 200 m races in backstroke, breaststroke, and butterfly techniques, while additional races for freestyle were held in 400 m and 800 m distances. From each swimmer, the year of competition, first name, last name, age, age group, and distance were recorded. Age groups were formed by grouping individuals every five years starting from the age of 25, ranging from 25 to 29, 30–34, 35–39, 40–44, 45–49, 50–54, 55–59, 60–64, 65–69, 70–74, 75–79, to 80 years or older (80+) (www.worldaquatics.com/masters/latest).

### Data cleaning

Following extraction from the World Aquatics archive, the dataset underwent a structured cleaning protocol. (i) Duplicates+defined as concurrent matches on year, first name, last name, age group, stroke, and distance—were identified and removed. (ii) Entries with missing, non-numeric, or zero race times were excluded from performance analyses but retained in participation counts when year and nationality were otherwise valid. (iii) Race times exceeding three standard deviations above the event-specific mean (per stroke, distance, and sex) were flagged for manual review against the source archive. In all but one instance, these flagged values were confirmed as physiologically plausible age-group performances (notably among the oldest age brackets) and retained. The single exception—a 1903.00 s entry in the men’s 50 m butterfly—was reviewed against the official World Aquatics archive and confirmed as a recording artifact at the source; it was retained in the dataset for full transparency, but its exclusion does not alter any reported mean or statistical comparison (see Table 2 footnote). (iv) Nationality abbreviations were standardized against the IOC three-letter country-code list. The recoding of “NIA” entries to “RUS” for the 2023 and 2024 editions, and the bounded impact of this recoding on national rankings, is detailed in the preceding paragraph.

### Statistical analysis

Descriptive data were presented using mean, standard deviation, maximum and minimum values, and confidence intervals. Overall, the top-ten race times for each swimming stroke, distance, and sex were identified for descriptive purposes. The top-ten times were selected to provide a robust and consistent sample of high-performance outcomes, balancing statistical power with performance homogeneity. This cutoff was chosen based on (1) observed time gaps in preliminary heats, (2) the need for sufficient sample size across age groups, and (3) alignment with prior research demonstrating that Masters-level performance often clusters in broader groupings than elite competitions. The unit of analysis was defined as swimmer-year entries, representing individual performance records within a specific competition year. As unique swimmer identifiers were not consistently available in the database, individual athletes may contribute multiple entries across years and events. Each entry is treated as an independent record, though this assumption may inflate the representation of prolific competitors. The top-ten cutoff was pre-defined to focus on high-performance outcomes and ensure a homogeneous sample of elite-level results. This approach maintains data quality, supports practical comparability across divisions, and is consistent with established practices in sports science research. The focus of this design is elite-level dominance and is not intended to characterize the broader Masters swimming population. To provide a structured analysis of competitive dominance, nationalities were grouped a priori into six categories. This included the five nations with the highest frequency of appearances in the annual top-ten for each specific stroke, distance, and sex, while all remaining nations were consolidated into a single ‘Others’ category. The categorization strategy was designed to isolate the primary drivers of elite performance while maintaining the ‘Others’ group as a necessary global benchmark for comparison. This ensured a clearer understanding of trends among the most prominent nationalities while also providing comprehensive coverage of all participating countries. The segmentation is done for convenience, aiming to identify which nationalities have the highest likelihood of podium placement.

Performance data were analyzed by aggregating all age groups within each sex and event. This aggregate approach was chosen to evaluate the overall competitive impact and the breadth of talent of a nation within the World Masters Championships, reflecting its global standing in the sport. Consequently, no age-adjustment or standardization was applied to the national rankings. The data did not follow a normal distribution or exhibit homogeneous variances, as determined by Shapiro-Wilk and Levene’s tests. Therefore, the Kruskal-Wallis test was used to compare differences between nationalities, and multiple pairwise comparisons adjusted by Bonferroni correction were performed to identify differences.

To control for family-wise error rates across the various comparisons, multiple pairwise comparisons were strictly adjusted using the Bonferroni correction. To quantify the magnitude of the differences observed between nationalities, effect sizes were calculated for the Kruskal-Wallis tests and reported as epsilon squared (ε^2^). The independence of observations was based on the treatment of each entry as a unique performance record within its specific context. Success was operationally defined as the frequency of a nation’s appearances in the annual top-ten fastest times for each stroke, distance, and sex; mean race times are reported as complementary descriptive statistics. Bonferroni correction was applied within each omnibus Kruskal-Wallis test to adjust pairwise comparisons for family-wise error. No additional correction was imposed across omnibus tests. This decision reflects the study’s design: the primary outcome—cumulative frequency of top-ten appearances per nation—is a descriptive tabulation that does not depend on significance testing, and the Kruskal-Wallis tests serve a secondary, contextualizing role. Pairwise comparisons, which underpin the specific between-nation claims made in the Results, are conservatively corrected within each test. Applying a further correction across omnibus tests in an exploratory descriptive context would compound conservatism in ways that have been argued against in the methodological literature [29]. Readers are therefore encouraged to interpret p-values in conjunction with the reported effect sizes (epsilon-squared) and confidence intervals, which provide the substantive basis for inference.

The significance level was set at 0.05, and SPSS version 26.0 (SPSS, Inc., Chicago, IL, USA) was used for all statistical analyses. While the hierarchical structure of the data (performances nested within swimmers, events, and years) would in principle favor multilevel modeling, the absence of reliable unique swimmer identifiers and the descriptive focus of this study precluded such an approach. Future studies with linked individual-level data should consider mixed-effects models.

The absence of reliable unique swimmer identifiers in the World Aquatics Masters archive precludes adjustment for repeated measurements of the same athletes across years and events. We treat each swimmer-year entry as an independent observation within its specific event-year-stroke-distance context. This assumption may inflate the representation of prolific competitors at the aggregate level, but we note that (i) the primary outcome—cumulative frequency of top-ten appearances—measures exactly the construct of sustained national representation, for which repeated appearances by the same elite athletes are substantively informative rather than nuisance variance; and (ii) the use of non-parametric Kruskal-Wallis tests avoids distributional assumptions that would be violated under repeated-measures correlation. A proper hierarchical analysis (performances nested within swimmers, events, and years) would require individual-level identifiers not available in the public archive and remain an important direction for future work.

Two common adjustments were deliberately not applied, and we justify their omission here. First, no adjustment was made for national participation rates. National participation is a constitutive component of the “elite national representation” construct measured by our primary outcome; conditioning on participation would estimate a counterfactual that is orthogonal to our research question. Second, no age-standardization was applied to national rankings. Our sample is restricted to the top-ten swimmer-year entries per event, a subset already filtered on competitive performance; within this elite stratum, the joint distribution of national origin and age is itself substantively informative rather than a confounder. All participation summaries are nevertheless stratified by sex and five-year age group to allow the reader to inspect demographic composition directly (see Results and Table 1).

**Table 1:** Participation distribution by stroke and distance (1986–2024).

Stroke	Distance	Total (N)	Women (N)	Men (N)	%
**Backstroke**	50 m	13111	6779	6332	39.8
	100 m	10611	5585	5026	32.2
	200 m	9209	4866	4343	28.0
**Breaststroke**	50 m	17575	7911	9664	40.7
	100 m	15211	7158	8053	35.2
	200 m	10373	4934	5439	24.0
**Butterfly**	50 m	15531	6361	9170	53.5
	100 m	8170	3469	4701	28.1
	200 m	5324	2436	2888	18.3
**Freestyle**	50 m	25094	10909	14185	30.6
	100 m	20961	8796	12165	25.6
	200 m	14309	6729	7580	17.4
	400 m	10956	5363	5593	13.4
	800 m	10317	5179	5138	12.6

N (total number); Data sourced from World Aquatics archives.

### Ethics statement

This study was approved by the Institutional Review Board of Kanton St. Gallen, Switzerland, with a waiver of the requirement for informed consent of the participants as the study involved the analysis of publicly available data (EKSG 01/06/2010). The study was conducted in accordance with recognized ethical standards according to the Declaration of Helsinki adopted in 1964 and revised in 2013.

## Results

National success was assessed primarily by the frequency of appearances among the annual top-ten fastest times; mean race times are reported as complementary descriptive statistics. Participation trends across all Masters swimming disciplines between 1986 and 2024 were analyzed using the complete dataset of 204,005 swimmer-year performance entries. Each entry, representing an individual performance record (swimmer-year), serves as the unit of analysis for these descriptive statistics. Participation percentages derived from this total cohort show that most swimmers competed in 50 m events: backstroke (39.8%), breaststroke (40.7%), butterfly (53.5%), and freestyle (30.6%). The second most common distance was 100 m (backstroke: 32.2%; breaststroke: 35.2%; butterfly: 28.1%; freestyle: 25.6%), followed by 200 m races (backstroke: 28.0%; breaststroke: 24.0%; butterfly: 18.3%; freestyle: 17.4%). In freestyle, additional participation occurred in 400 m (13.4%) and 800 m (12.6%) events. For a comprehensive overview of participation distribution and relevant denominators for each category, please refer to Table 1.

Regarding performance in 50 m events, freestyle swimmers recorded the fastest overall mean time (34.56 ± 10.0 s), followed by butterfly (36.21 ± 18.28 s), backstroke (43.55 ± 13.08 s), and breaststroke (44.37 ± 12.19 s), as detailed in Table 2. While 100 m and 200 m races exhibited similar performance patterns, with men consistently faster than women across all strokes.

**Table 2:** Fastest 50 m mean race times by stroke (1986–2024).

Stroke	Overall Mean (s) ± SD	Min (s)	Max (s)	Female Mean (s) ± SD	Female Min/Max (s)	Male Mean (s) ± SD	Male Min/Max (s)
**Freestyle**	34.56 ± 10.00	22.94	156.18	39.16 ± 11.74	25.73/ 156.18	31.02 ± 6.48	22.94/ 137.68
**Butterfly**	36.21 ± 18.28	24.11	1903.00†	40.74 ± 12.34	27.10/ 198.35	33.07 ± 20.88	24.01/ 1903.00†
**Backstroke**	43.55 ± 13.08	25.92	154.38	48.01 ± 13.95	28.85/ 154.38	38.77 ± 10.09	25.92/ 142.15
**Breaststroke**	44.37 ± 12.19	27.65	209.77	50.00 ± 13.73	31.99/ 209.77	39.76 ± 8.29	27.65/ 159.54

Values expressed in seconds; SD (Standard deviation) † This value likely reflects a recording error in the original World Aquatics archive; its exclusion does not alter reported means or statistical comparisons; Min (minimum); Max (maximum); Data sourced from World Aquatics archives.

Sex distribution varied across events. In most events, men outnumbered women, except in the 50 m backstroke, where participation was nearly equal. The 800 m freestyle event had the most balanced gender participation, with 5,179 women and 5,138 men. To ensure that variations in age distribution between sexes did not confound the observed participation trends, all data were stratified by both sex and five-year age groups across all events. This stratification allows for a clear interpretation of participation and performance within each specific demographic category, ensuring that findings are not obscured by differences in the age profiles of the male and female cohorts.

For comparative analysis of national performances, the five countries with the highest representation in the annual top-ten fastest times for each distance and gender were identified. Germany led in female breaststroke, butterfly, and 50 m backstroke. Brazilian men excelled in 50 m backstroke, Russian men in 50 m breaststroke, and U.S. swimmers dominated most other distances, particularly men’s butterfly, breaststroke (100 m and 200 m), all backstroke distances (except 50 m), and all freestyle events. The other countries were grouped into a single category called “Others.” The mean times of all top-ten swimmers by year for each sex and country were compared and are displayed by sex in Tables 3, 5, 7, and 9 among women, and Tables 4, 6, 8, and 10 among men for all the Masters swimming disciplines. Germany was the country with the highest number of female swimmers in the top-ten times by year for all the breaststroke (Table 5) and butterfly (Table 7) distances, as well as for the 50 m backstroke style (Table 3). The number of Brazilian male swimmers led in the 50 m backstroke distance (Table 4), along with Russian male athletes in the 50 m breaststroke distance (Table 6). In all these competitions, U.S. swimmers were in second place, except for females in the 50 m breaststroke, where they finished in third place (Tables 3–5,7). However, the country that most frequently reached the top-ten per year for all the butterfly competitions among men (Table 8) and for the 100 m and 200 m breaststroke races (Table 6), as well as for all 100 m and 200 m backstroke races (Tables 3, 4) and for all freestyle distances (Tables 9, 10), was the USA. Other countries that mostly made it into the top-ten were Italy in men’s backstroke and breaststroke, and above all, Great Britain in all disciplines.

**Table 3:** Characteristics of differences between nationalities in swimming mean times for female athletes in the top 10 times in all backstroke distances.

Race	Country		Mean ± SD	CI 95%	Min/ Max	χ2	Df	P	ϵ^2^	Post-hoc differences
**50 m Backstroke**	USA	26	31.97 ± 1.25	31.46 - 32.48	25.85/ 34.42	4,183	5	0.394	0.021	
	Others	95	32.33 ± 1.17	32.10 - 32.57	29.89/ 35.54					
	JPN	16	32.51 ± 1.79	31.55 - 33.46	29.74/ 35.97					
	GBR	21	32.53 ± 0.84	32.15 - 32.91	31.14/ 34.79					
	GER	27	32.63 ± 0.71	32.35 - 32.91	31.12/ 33.75					
	BRA	15	32.86 ± 1.56	32.00 - 33.72	30.20/ 35.74					
**100 m Backstroke**	JPN	14	69.51 ± 2.89	67.84 - 71.17	65.43/ 75.24	9,697	5	0.084	0.049	
	USA	36	69.64 ± 3.57	68.43 - 70.84	61.75/ 78.47					
	Others	96	69.85 ± 2.93	69.26 - 70.44	64.00/ 78.78					
	GBR	20	70.66 ± 2.89	69.31 - 72.01	67.36/ 77.30					
	BRA	12	70.77 ± 2.67	69.07 - 72.47	67.41/ 77.06					
	GER	22	71.04 ± 1.72	70.28 - 71.81	67.88/ 74.83					
**200 m Backstroke**	ITA	11	151.06 ± 4.34	148.14 - 153.98	143.93/ 160.12	8,858	5	0.115	0.045	
	USA	41	152.33 ± 6.22	150.37 - 154.30	139.98/ 169.99					
	Others	92	153.85 ± 8.54	152.08 - 155.62	121.91/ 175.17					
	GBR	31	153.88 ± 7.94	150.97 - 156.79	136.28/ 171.19					
	GER	14	155.43 ± 4.10	153.06 - 157.80	147.23/ 160.44					
	BRA	11	155.50 ± 4.39	152.55 - 158.45	150.37/ 163.17					

Values expressed in seconds. N (total sample size per nation); SD (Standard deviation); CI (Confidence Interval). Epsilon squared (**ϵ**^**2**^) represents the effect size for the Kruskal-Wallis test. All p-values for pairwise comparisons (not shown) were adjusted using the Bonferroni correction. Data sourced from World Aquatics archives.

**Table 4:** Characteristics of differences between nationalities in swimming mean times for male athletes in the top 10 times in all backstroke distances.

Race	Country		Mean ± SD	CI 95%	Min/ Max	χ2	Df	P	ϵ^2^	Post-hoc differences
**50 m Backstroke**	JPN	11	27.32 ± 1.37	26.40 - 28.24	26.27/ 30.97	12,876	5	0.025	0.068	BRA vs JPN
	Others	88	28.23 ± 1.25	27.96 - 28.49	25.92/ 30.67					
	BRA	37	28.29 ± 1.35	27.84 - 28.74	26.44/ 30.92					
	ITA	13	28.29 ± 0.99	27.68 - 28.89	27.15/ 30.70					
	GER	13	28.35 ± 0.80	27.86 - 28.83	26.81/ 29.71					
	USA	28	28.73 ± 1.01	28.34 - 29.13	26.72/ 30.49					
**100 m Backstroke**	Others	88	60.69 ± 3.03	60.05 - 61.34	49.13/ 67.59	15,575	5	0.008	0.082	Others vs USA
	ITA	13	61.07 ± 1.10	60.41 - 61.73	59.53/ 63.21					
	BRA	28	61.94 ± 2.54	60.11 - 62.08	57.53/ 67.60					
	GER	14	61.98 ± 2.19	60.72 - 63.25	58.09/ 66.83					
	USA	32	62.62 ± 2.67	61.65 - 63.58	58.66/ 68.29					
	GBR	15	62.73 ± 3.06	61.03 - 64.42	57.49/ 67.76					
**200 m Backstroke**	FRA	17	134.93 ± 4.40	132.67 - 137.19	128.23/ 145.79	7,257	5	0.202	0.038	
	GER	22	135.07 ± 4.64	133.01 - 137.13	123.80/ 145.07					
	ITA	14	135.79 ± 5.18	132.80 - 138.78	129.67/ 149.64					
	Others	90	135.81 ± 6.13	134.52 - 137.09	123.62/ 152.01					
	BRA	16	136.37 ± 5.26	133.57 - 139.17	130.24/ 151.71					
	USA	31	137.82 ± 4.64	136.12 - 139.52	125.62/ 147.66					

Values expressed in seconds. N (total sample size per nation); SD (Standard deviation); CI (Confidence Interval). Epsilon squared (**ϵ**^**2**^) represents the effect size for the Kruskal-Wallis test. All p-values for pairwise comparisons (not shown) were adjusted using the Bonferroni correction. Data sourced from World Aquatics archives.

**Table 5:** Characteristics of differences between nationalities in swimming mean times for female athletes in the top 10 times in all breaststroke distances.

Race	Country		Mean ± SD	CI 95%	Min/ Max	χ2	Df	P	ϵ^2^	Post-hoc differences
**50 m Breaststroke**	GER	48	34.78 ± 0.16	34.45 - 35.11	32.66/ 37.44	22,272	5	<0.001	0.112	RUS vs Others/ITA/USA/GBR GER vs Others/USA/GBR
	Others	81	35.47 ± 0.18	35.11 - 35.83	31.99/ 39.87					
	ITA	14	35.55 ± 0.35	34.80 - 36.30	33.69/ 38.15					
	USA	21	35.75 ± 0.37	34.97 - 36.52	33.33/ 38.37					
	GBR	26	35.91 ± 0.22	35.46 - 36.36	33.90/ 37.86					
	RUS	10	36.75 ± 0.37	32.91 - 34.59	32.03/ 35.53					
**100 m Breaststroke**	GER	46	77.16 ± 0.42	76.31 - 78.00	73.13/ 83.88	15,604	5	0.008	0.078	GER vs USA/GBR ITA vs GBR Others vs USA/GBR
	ITA	11	77.25 ± 0.76	75.56 - 78.93	74.41/ 81.57					
	Others	77	78.82 ± 1.18	76.46 - 81.17	71.01/ 163.56					
	AUS	9	79.11 ± 1.15	76.47 - 81.75	73.12/ 83.67					
	USA	29	79.50 ± 0.70	78.06 - 80.94	73.82/ 86.31					
	GBR	28	79.74 ± 0.63	78.45 - 81.04	74.71/ 86.77					
**200 m Breaststroke**	GER	45	168.15 ± 0.98	166.18 - 170.12	157.68/ 179.24	13,796	5	0.017	0.069	GER vs Others/USA/GBR
	FRA	8	168.40 ± 1.69	164.41 - 172.39	161.03/ 177.06					
	ITA	11	169.48 ± 1.79	165.49 - 173.47	161.07/ 177.73					
	Others	79	170.86 ± 0.81	169.24 - 172.48	155.90/ 191.72					
	USA	28	172.72 ± 1.50	169.64 - 175.79	158.44/ 187.35					
	GBR	29	174.25 ± 1.40	171.39 - 177.12	162.90/ 192.26					

Values expressed in seconds. N (total sample size per nation); SD (Standard deviation); CI (Confidence Interval). Epsilon squared (**ϵ**^**2**^) represents the effect size for the Kruskal-Wallis test. All p-values for pairwise comparisons (not shown) were adjusted using the Bonferroni correction. Data sourced from World Aquatics archives.

**Table 6:** Characteristics of differences between nationalities in swimming mean times for male athletes in the top 10 times in all breaststroke distances.

Race	Country		Mean ± SD	CI 95%	Min/ Max	χ2	Df	P	ϵ^2^	Post-hoc differences
**50 m** **Breaststroke**	JPN	17	29.48 ± 0.47	28.49 - 30.47	27.83/ 32.99	28,519	5	<0.001	0.151	JPN vs Others/GER/USA RUS vs Others/GER/USA ITA vs Others/GER/USA
	RUS	23	29.54 ± 0.19	29.14 - 29.93	27.84/ 31.23					
	ITA	15	29.69 ± 0.18	29.31 - 30.07	28.35/ 30.54					
	GER	19	30.51 ± 0.18	30.12 - 30.89	28.88/ 31.38					
	Others	96	30.56 ± 0.12	30.32 - 30.80	27.65/ 32.83					
	USA	20	30.82 ± 0.23	30.34 - 31.31	29.01/ 32.78					
**100 m Breaststroke**	RUS	19	66.21 ± 0.53	65.08 - 67.33	63.16/ 71.65	16,807	5	0.005	0.089	RUS vs Others/GBR/GER/USA ITA vs Others/GER/USA
	ITA	17	66.56 ± 0.31	65.91 - 67.21	64.43/ 68.43					
	GBR	16	68.19 ± 0.61	66.89 - 69.49	64.11/ 70.83					
	Others	88	68.36 ± 0.37	67.62 - 69.10	61.77/ 80.88					
	GER	18	68.43 ± 0.40	67.59 - 69.27	64.67/ 70.80					
	USA	32	68.73 ± 0.42	67.87 - 69.60	64.58/ 74.33					
**200 m Breaststroke**	RUS	16	146.54 ± 1.09	144.20 - 148.87	139.45/ 155.10	27,471	5	<0.001	0.145	RUS vs Others/USA/GER ITA vs Others/GER/USA GBR vs GER/USA Others vs USA/GER
	ITA	15	147.03 ± 0.92	145.06 - 149.00	141.16/ 152.00					
	GBR	15	149.87 ± 1.51	146.62 - 153.12	140.29/ 157.89					
	Others	97	150.88 ± 0.71	149.47 - 152.29	136.79/ 165.44					
	GER	23	154.02 ± 0.81	152.34 - 155.70	146.37/ 161.22					
	USA	24	154.93 ± 1.51	151.81 - 158.04	141.20/ 170.32					

Values expressed in seconds. N (total sample size per nation); SD (Standard deviation); CI (Confidence Interval). Epsilon squared (**ϵ**^**2**^) represents the effect size for the Kruskal-Wallis test. All p-values for pairwise comparisons (not shown) were adjusted using the Bonferroni correction. Data sourced from World Aquatics archives.

**Table 7:** Characteristics of differences between nationalities in swimming mean times for female athletes in the top 10 times in all butterfly distances.

Race	Country		Mean ± SD	CI 95%	Min/ Max	χ2	Df	P	ϵ^2^	Post-hoc differences
**50 m Butterfly**	RUS	14	29.04 ± 0.25	28.49 - 29.59	27.46/ 30.74	13,063	5	0.023	0.065	RUS vs Others/GER/USA/GBR Others vs GBR
	Others	90	29.87 ± 0.13	29.62 - 30.13	27.75/ 33.36					
	GER	32	29.95 ± 0.17	29.60 - 30.30	28.19/ 31.71					
	USA	31	30.12 ± 0.24	29.63 - 30.60	27.46/ 32.59					
	JPN	13	30.27 ± 0.48	29.13 - 31.31	28.72/ 33.28					
	GBR	20	30.35 ± 0.26	29.81 - 30.89	27.10/ 32.52					
**100 m Butterfly**	ITA	18	66.85 ± 0.42	65.97 - 67.73	63.37/ 70.11	6,528	5	0.258	0.032	
	GER	36	67.09 ± 0.31	66.46 - 67.72	62.94/ 70.18					
	Others	81	67.52 ± 0.36	66.81 - 68.23	59.99/ 78.10					
	USA	33	67.53 ± 0.50	66.52 - 68.54	63.18/ 76.17					
	GBR	24	68.57 ± 0.68	67.16 - 69.99	61.93/ 76.65					
	NED	8	69.69 ± 1.44	66.28 - 73.10	65.47/ 77.89					
**200 m** **Butterfly**	GER	45	150.53 ± 0.91	148.70 - 152.36	137.72/ 170.51	13,248	5	0.021	0.066	
	ITA	14	150.97 ± 1.07	148.67 - 153.27	141.46/ 158.98					
	GBR	23	151.67 ± 1.46	148.65 - 154.69	140.72/ 176.25					
	USA	29	153.93 ± 1.88	150.08 - 157.77	142.80/ 190.57					
	Others	80	155.12 ± 1.31	152.52 - 157.73	128.11/ 199.65					
	AUS	9	159.75 ± 4.63	149.07 - 170.44	151.48/ 195.08					

Values expressed in seconds. N (total sample size per nation); SD (Standard deviation); CI (Confidence Interval). Epsilon squared (**ϵ**^**2**^) represents the effect size for the Kruskal-Wallis test. All p-values for pairwise comparisons (not shown) were adjusted using the Bonferroni correction. Data sourced from World Aquatics archives.

**Table 8:** Characteristics of differences between nationalities in swimming mean times for male athletes in the top 10 times in all butterfly distances.

Race	Country		Mean ± SD	CI 95%	Min/ Max	χ2	Df	P	ϵ^2^	Post-hoc differences
**50 m** **Butterfly**	RUS	15	25.48 ± 0.15	25.13 - 25.79	24.75/ 26.60	15,157	5	0.010	0.076	RUS vs BRA/USA/GER Others vs USA
	Others	93	25.84 ± 0.08	25.68 - 26.00	24.11/ 27.65					
	GBR	13	25.93 ± 0.27	25.33 - 26.51	24.27/ 27.40					
	BRA	18	26.07 ± 0.18	25.70 - 26.45	24.96/ 27.74					
	USA	38	26.21 ± 0.11	25.98 - 26.44	24.34/ 27.84					
	GER	13	26.23 ± 0.13	25.95 - 26.51	25.63/ 27.21					
**100 m** **Butterfly**	Others	88	57.81 ± 0.18	57.45 - 58.16	54.12/ 63.05*	6,469	5	0.263	0.034	
	ITA	14	57.97 ± 0.39	57.13 - 58.81	56.15/ 61.30					
	BRA	20	58.21 ± 0.39	57.39 - 59.02	55.46/ 63.05					
	GBR	18	58.39 ± 0.39	57.57 - 59.22	56.35/ 62.19					
	GER	12	58.52 ± 0.36	57.73 - 59.32	56.69/ 61.30					
	USA	38	58.68 ± 0.33	58.02 - 59.35	54.02/ 62.73					
**200 m** **Butterfly**	FRA	13	133.14 ± 1.10	130.74 - 135.54	127.85/ 139.82	1,436	5	0.920	0.008	
	ITA	21	133.82 ± 0.76	132.24 - 135.40	127.92/ 141.09					
	GBR	14	134.02 ± 1.33	131.14 - 136.90	126.84/ 146.63					
	USA	30	134.76 ± 1.13	132.44 - 137.08	126.74/ 148.43					
	Others	92	134.96 ± 0.77	133.43 - 136.50	123.43/ 165.34					
	GER	20	135.33 ± 1.01	133.20 - 137.45	131.61/ 147.28					

Values expressed in seconds. N (total sample size per nation); SD (Standard deviation); CI (Confidence Interval). Epsilon squared (**ϵ**^**2**^) represents the effect size for the Kruskal-Wallis test. All p-values for pairwise comparisons (not shown) were adjusted using the Bonferroni correction. Data sourced from World Aquatics archives.

**Table 9:** Characteristics of differences between nationalities in swimming mean times for female athletes in the top 10 times all freestyle distances.

Race	Country	N	Mean ± SD	CI 95%	Min/ Max	χ2	Df	P	ϵ^2^	Post-hoc differences
**50 m** **Freestyle**	RUS	17	27.35 ± 0.14	27.06 - 27.64	26.02/ 28.03	13,022	5	0.023	0.065	RUS vs Others/USA/GER/GBR GBR vs Others/JPN/USA/GER
	GER	26	27.81 ± 0.15	27.50 - 28.11	26.10/ 28.78					
	USA	36	27.86 ± 0.12	27.61 - 28.10	26.67/ 29.53					
	Others	97	27.86 ± 0.09	27.68 - 28.04	25.99/ 30.45					
	JPN	10	27.90 ± 0.37	27.05 - 27.75	26.85/ 30.42					
	GBR	14	28.35 ± 0.28	27.75 - 29.95	25.73/ 30.05					
**100 m** **Freestyle**	GER	21	60.68 ± 0.37	59.91 - 61.46	57.74/ 64.14	5,169	5	0.396	0.026	
	CAN	13	60.78 ± 0.27	60.18 - 61.38	59.03/ 62.35					
	Others	96	60.95 ± 0.21	60.54 - 61.37	56.89/ 67.78					
	FRA	9	61.22 ± 0.49	60.09 - 62.35	59.64/ 64.12					
	USA	43	61.29 ± 0.30	60.68 - 61.90	58.65/ 67.49					
	GBR	18	61.85 ± 0.56	60.67 - 63.03	56.96/ 65.93					
**200 m** **Freestyle**	CAN	9	131.75 ± 0.67	130.20 - 133.30	129.12/ 135.92	10,132	5	0.072	0.051	
	GER	23	132.83 ± 0.70	131.38 - 134.27	125.76/ 138.09					
	FRA	8	133.36 ± 1.21	130.50 - 136.22	129.97/ 139.55					
	Others	93	133.46 ± 0.48	132.52 - 134.41	122.35/ 149.15					
	USA	45	134.01 ± 0.66	132.69 - 135.34	126.91/ 149.20					
	GBR	22	136.02 ± 1.15	133.63 - 138.41	125.97/ 148.20					
**400 m** **Freestyle**	GER	30	275.32 ± 4.94	265.21 - 285.43	137.72/ 299.27	6,203	5	0.287	0.033	
	GBR	20	277.19 ± 7.84	260.76 - 293.61	136.28/ 320.01					
	Others	77	279.31 ± 2.96	273.40 - 285.21	133.36/ 321.35					
	ITA	9	281.27 ± 1.07	278.80 - 283.74	275.19/ 286.45					
	FRA	9	281.92 ± 1.60	278.22 - 285.62	276.37/ 292.75					
	USA	45	285.50 ± 1.52	282.43 - 288.57	266.50/ 318.49					
**800 m** **Freestyle**	ITA	17	582.07 ± 2.54	576.69 - 587.45	565.74/ 602.96	5,468	5	0.361	0.027	
	GER	25	584.44 ± 3.81	576.57 - 592.30	547.57/ 621.48					
	Others	81	587.14 ± 2.70	581.76 - 592.52	540.00/ 677.53					
	GBR	21	590.42 ± 5.75	578.42 - 602.42	538.94/ 652.35					
	USA	48	592.10 ± 3.43	585.19 - 599.00	553.49/ 667.59					
	AUS	8	615.40 ± 14.57	580.95 - 649.86	565.97/ 658.21					

Values expressed in seconds. N (total sample size per nation); SD (Standard deviation); CI (Confidence Interval). Epsilon squared (**ϵ**^**2**^) represents the effect size for the Kruskal-Wallis test. All p-values for pairwise comparisons (not shown) were adjusted using the Bonferroni correction. Data sourced from World Aquatics archives.

**Table 10:** Characteristics of differences between nationalities in swimming mean times for male athletes in the top 10 times in all freestyle distances.

Race	Country	N	Mean ± SD	CI 95%	Min/ Max	χ2	Df	P	ϵ^2^	Post-hoc differences
**50 m** **Freestyle**	RUS	13	23.92 ± 0.15	23.58 - 24.25	23.12/ 25.31	12,099	5	0.033	0.064	RUS vs GBR/USA Others vs USA BRA vs USA
	BRA	27	24.14 ± 0.11	23.92 - 24.36	23.24/ 25.07					
	Others	84	24.14 ± 0.07	24.00 - 24.28	22.94/ 25.47					
	GER	18	24.21 ± 0.10	24.01 - 24.42	23.64/ 25.12					
	GBR	13	24.37 ± 0.16	24.02 - 24.71	23.73/ 25.52					
	USA	35	24.42 ± 0.08	24.25 - 24.58	23.27/ 25.30					
**100 m** **Freestyle**	ITA	11	53.09 ± 0.32	52.38 - 53.80	51.05/ 54.50	10,164	5	0.071	0.054	
	Others	82	53.30 ± 0.14	53.03 - 53.57	50.88/ 56.43					
	GER	15	53.45 ± 0.32	52.77 - 54.13	51.75/ 55.95					
	BRA	22	53.53 ± 0.28	52.94 - 54.12	51.31/ 56.91					
	GBR	16	53.64 ± 0.35	52.89 - 54.39	52.05/ 56.65					
	USA	44	53.99 ± 0.18	53.62 - 54.35	51.56/ 56.60					
**200 m** **Freestyle**	Others	82	118.92 ± 0.38	118.17 - 119.67	110.76/ 128.29	13,931	5	0.016	0.074	Others vs USA/BRA
	ITA	14	119.40 ± 0.53	118.25 - 120.56	117.43/ 125.46					
	GER	19	119.55 ± 0.53	118.44 - 120.65	115.68/ 124.86					
	FRA	15	120.66 ± 0.67	119.22 - 122.10	117.75/ 125.24					
	USA	48	120.69 ± 0.59	119.50 - 121.87	112.84/ 128.88					
	BRA	12	121.21 ± 0.54	120.03 - 122.39	118.58/ 125.29					
**400 m** **Freestyle**	Others	82	256.04 ± 1.90	252.26 - 259.81	121.91/ 283.30	25,853	5	0.001	0.144	ITA vs USA/BRA Others vs USA/BRA FRA vs USA GER vs USA
	ITA	23	256.07 ± 1.33	253.30 - 258.84	244.17/ 269.99					
	FRA	12	257.93 ± 1.53	254.57 - 261.30	253.50/ 272.88					
	GER	17	258.75 ± 1.27	256.05 - 261.44	250.78/ 269.06					
	USA	37	263.00 ± 1.16	260.65 - 265.36	248.70/ 280.34					
	BRA	9	263.65 ± 2.55	257.78 - 269.52	256.97/ 276.70					
**800 m** **Freestyle**	ESP	10	527.00 ± 2.51	521.32 - 532.68	518.86/ 542.93	32,914	5	0.001	0.174	ESP vs Others/GER/USA/BRA ITA vs Others/GER/USA/BRA Others vs USA/BRA GER vs BRA
	ITA	30	531.29 ± 2.39	526.40 - 536.19	508.77/ 570.22					
	Others	81	537.46 ± 1.96	533.57 - 514.36	496.52/ 595.55					
	GER	21	538.91 ± 2.84	533.00 - 544.82	515.45/ 562.57					
	USA	36	545.77 ± 2.76	540.16 - 551.38	516.88/ 579.80					
	BRA	12	564.76 ± 7.75	547.72 - 581.81	522.85/ 605.84					

Values expressed in seconds. N (total sample size per nation); SD (Standard deviation); CI (Confidence Interval). Epsilon squared (**ϵ**^**2**^) represents the effect size for the Kruskal-Wallis test. All p-values for pairwise comparisons (not shown) were adjusted using the Bonferroni correction. Data sourced from World Aquatics archives.

**Fig 1:**
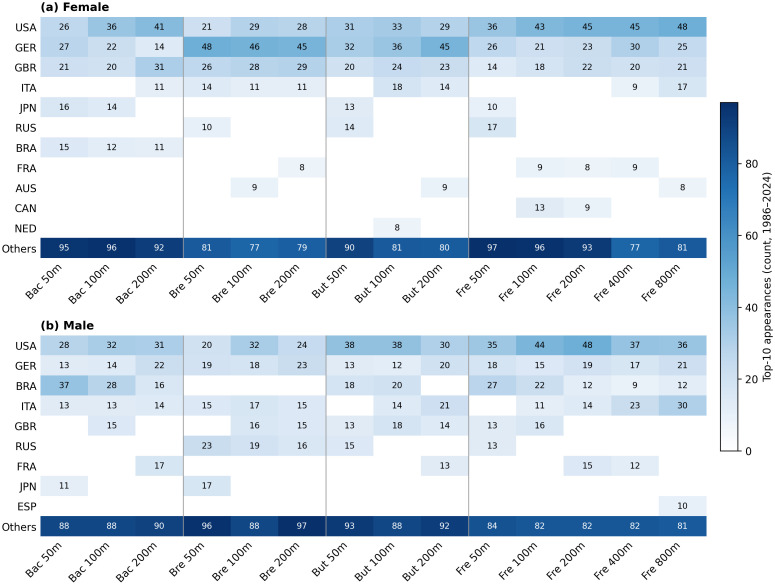
Top-ten appearance frequency by nation, stroke and distance at the World Aquatics Championships, 1986−2024. Rows: top five nations per sex plus “Others” (all other countries pooled). Columns: swimming events grouped by stroke (backstroke 50/100/200 m, breaststroke 50/100/200 m, butterfly 50/100/200 m, freestyle 50/100/200/400/800 m). Cells: cumulative count of top-10 appearances across 20 championship editions; color scale is shared between panels. Panel (a) female athletes; panel (b) male athletes. Bac (Backstroke); Bre (Breaststroke); But (Butterfly); Fre (Freestyle); Data sourced from World Aquatics archives.

A visual synthesis of these national representation patterns—top-ten appearance frequency by nation across all stroke × distance combinations, split by sex—is provided in Fig 1.

Significant differences in performance were found between the countries. Russian female swimmers were most numerous than Others, including Italy, the USA, and Great Britain among the top-ten in the 50 m breaststroke (Table 5), and had more participants in the top-ten for the 50 m butterfly and 50 m freestyle than Others, Germany, the USA, and Great Britain(Tables 7, 9). The number of male swimmers from Russia stood out in breaststroke with better performances in the 50m than Others, Germany, and the USA; in the 100 m than Others, Great Britain, Germany, and the USA; and for 200 m distances than Others, the USA, and Germany (Table 6). Additionally, Russian men were more strongly represented in the top-ten than athletes from Brazil, the USA, and Germany in the 50 m butterfly (Table 8), and performed competitively in the 50 m freestyle compared to Great Britain and the USA (Table 10). Brazilian athletes outnumbered Japanese swimmers in the men’s 50 m backstroke (Table 4), and had more top-ten finishers than the USA in the men’s 50 m freestyle (Table 10). The group of Italian female swimmers were better represented among the top-ten than the British in the 100 m breaststroke (Table 5), while the Italian men outnumbered Others, Germany, and the USA in all breaststroke distances (Table 6), had more top-ten finishers than the USA and Brazil in the 400 m freestyle (Table 10), and also had more top-ten finishers than Others, Germany, the USA, and Brazil in the 800 m freestyle (Table 10). German female swimmers were most frequently among the top-ten, especially in breaststroke, with better performances in the 50 m than Others, Italy, the USA, and Great Britain; in the 100 m than the USA and Great Britain; and even in the 200 m distances than Others, the USA and Great Britain (Table 5). Similarly, the number of German male swimmers stood out in comparison to the USA in the 400 m freestyle, and to Brazil in the 800 m freestyle (Table 10). British female athletes were better represented among the leading finishers in the 50 m freestyle than their rivals from Japan, the USA and Germany (Table 9). Meanwhile, their male counterparts outnumbered the swimmers from Germany and the USA in the 200 m breaststroke (Table 6). Japanese male swimmers had more top-ten performers than Others, Germany, and the USA in the 50 m breaststroke (Table 6), while French male swimmers were more numerous than those from the USA in the 400 m freestyle (Table 10). Spanish male swimmers also had a larger group of top-ten athletes than Others, Germany, the USA, and Brazil in the 800 m freestyle (Table 10). Female swimmers summarized in the group of Others performed better than those from the USA and Great Britain in the 100 m breaststroke (Table 5), and achieved better results than those from Great Britain in the 50 m butterfly (Table 7); while the group of Others among men presented more top-ten swimmers compared to the USA in the 100 m backstroke and 50 m freestyle (Tables 4, 10), were more successful than swimmers from USA and Germany in the 200 m breaststroke (Table 6), performed better than the USA in the 50 m butterfly (Table 8), and achieved more top-ten rankings than the USA and Brazil in both the 200 m and 400 m freestyle (Table 10). No numerical superiority from a particular nation was observed in all other distances across the four disciplines in women’s and men’s competitions (Tables 3–10).

## Discussion

The main findings of our study were (i) the country with the highest number of female swimmers in the top-ten fastest times per year in all breaststroke and butterfly distances was Germany, (ii) the country with the highest number of male swimmers in the top-ten fastest times per year in all butterfly distances was the USA, (iii) Germany had the highest number of female swimmers for 50m backstroke, and Brazil had the highest number of male swimmers for 50 m backstroke, while the USA was in second place for both categories and had the highest number of female and male participants in all other backstroke distances, (iv) Russia had the highest number of male swimmers for 50 m breaststroke, while the USA was second in this category and had the highest number of male participants in all other breaststroke distances, (v) the USA had the highest number of female and male participants in all freestyle distances, (vi) the countries that were most often in the top-ten best times in all the World Masters Championships were the USA, Germany, Great Britain, Russia, Italy, and Brazil. Thus, the comparison of the results confirmed our initial hypothesis in large parts of our study, especially regarding U.S. male athletes.

Even though female swimmers from Germany had the highest number in the top-ten fastest times per year in all breaststroke and butterfly distances, as well as for 50 m backstroke, U.S. women followed each time closely behind by their successful competitors. This success of German female athletes comes as no surprise and has already been partially proven by recent studies on butterfly [24], backstroke [25], and freestyle [26] at the Masters Championships. Germany is known for its well-organized and comprehensive swimming infrastructure through the DSV (Deutscher Schwimm-Verband), which offers a long list of clubs and races for all age group swimmers (www.dsv.de/masterssport). German women, in particular, look back on a long tradition of Olympic champions (www.nbcolympics.com), despite the dark time of systematic doping of female swimmers during the GDR (German Democratic Republic) era (One of the most notorious examples of state-sponsored doping in sports history occurred in the GDR or East Germany, between the 1960s and 1980s. Under the direction of government authorities and the state security service (Stasi), a covert program known as State Plan 14.25 systematically administered performance-enhancing substances—primarily anabolic steroids—to athletes, including underage female swimmers. These substances were often given without the athletes’ informed consent, as part of a broader effort to elevate East Germany’s international status through sustained success in competitive sports) [30]. Previous studies have also shown that German athletes, in general, dominate participation and performance in endurance events such as the Ironman triathlon [31].

In the 50 m backstroke and 50 m breaststroke events, Brazilian and Russian male swimmers, respectively, achieved the highest levels of success; however, the number of male athletes from the United States consistently secured second place in both disciplines. Thus, large countries with substantial swimming associations (www.cbda.org.br, www.russwimming.ru) and a high number of athletes seem to play a decisive role in success [32]. The success of Brazilian swimmers also aligns closely with the Brazilian Olympic strategy for sports competition and medal achievement. Brazilian athletes have gained more medals in every Olympic cycle. Additionally, there may be a cultural increase in participation in Masters sports, particularly in swimming [33].

Competitors from several other countries, including Great Britain and Italy, were among the top-performing swimmers and, together with athletes from the USA, Germany, Brazil, and Russia, formed the group of the “Big Six”. However, the athletes with the most outstanding success, which were mostly under the top-ten most times per year in almost all male competitions, were primarily U.S. swimmers.

It remains an interesting phenomenon that athletes originating from a specific geographic region or country dominate certain sports disciplines [34]. International success in sports has been theorized to be, at least in part, determined by a set of variables that can be organized into different domains. These include, for example, financial support, competition participation, access to sports facilities, coaching development, and talent identification programs for “ideal demographic conditions” [19]. In sports science, elite performance can, therefore, be understood as a complex interplay between intrinsic and extrinsic factors, including an athlete’s environment, access to training, physiology, and genetic factors [35]. Although not fully understood, specific populations preserve a genetic pool that may predispose them to certain sporting advantages [10,11].

Genetic factors have been previously linked to several athletic phenotypes, which include height, muscle volume, muscle fiber type, fatty tissue distribution, lung volume, heart configuration, and, secondly, physical abilities like muscle function, lung capacity, cardiac output, and VO2max [36]. In terms of endemic populations of elite athletes, we already know that, for instance, the region of East Africa produces a number of elite long-distance runners since most national and international Kenyan athletes come from the Rift Valley province [8,9], and Ethiopian marathoners mainly originate from the regions of Arsi and Shewa [10]. However, the predisposition to athletic potential is only one part of athlete development, which includes recognizing, training, and promoting the physical skills and talents [37].

Climate and geography are external factors that significantly influence participation in open-water swimming competitions, where athletes must swim in natural bodies of water such as rivers, lakes, and oceans [12,20]. Countries with coastlines or easy access to such environments often have populations that engage in water-based leisure and physical activities, which may contribute to higher participation and performance levels. Athletes from these regions—especially those with strong sports infrastructure and physical activity policies—are more likely to excel in open-water events. These competitions are uniquely affected by environmental conditions such as water temperature, tides, currents, and waves, all of which play a critical role in shaping race strategy and performance outcomes [20,38,39]. As a result, there is a notable correlation between geographic and climatic characteristics and the success of nations in open-water swimming [20,22].

Numerous empirical studies show that the population’s size and wealth are among the most essential socioeconomic determinants of success [40,32]. The bigger the population, the larger the pool from which talents may be recruited, and the greater the opportunities to organize training and competitions [32]. However, compared to China, which has 1.4 billion people [41], the USA’s population is significantly smaller: 335 million [42]. Even athletes from countries with smaller populations, as was shown (i.e., Germany and Italy) [43], were among the top-ten best times in all the World Masters Championships. However, a larger population from which athletes can be drawn seems beneficial but may only sometimes be crucial for producing successful athletes. More competitors offer more opportunities for a nation to finish among the top-ten. Wealthy countries can invest more in sports and elite sports, and individuals may participate in a broader number of sports while enjoying a higher living standard [32]. Greater wealth is associated with more leisure time, which creates more opportunities to participate in competitive sports [32]. Furthermore, greater wealth and a high GDP (gross domestic product) are connected to good infrastructure that provides easy access to well-maintained and high-quality swimming facilities, coaching expertise, and sports science support services [20].

Success in sports, on the other hand, promotes a “feel-good” factor among citizens and bestows prestige upon the nation [44]. Countries with a long and successful tradition in aquatic sports have extensive experience and a rich legacy [44]. U.S. athletes have been dominating Olympic swimming for decades [27,28], which serves as a motivating factor and could put pressure on current athletes to keep that legacy alive.

Also, the “fluidity” between professional and amateur swimming is questionable. So, it is striking that the findings observed in the present study regarding the performance of the “Big Six” match the all-time medal count of athletes from these countries at the Olympic Games. Particularly, the “Big Six,” except for Brazil, are listed among the top-ten countries for all-time Olympic medals (https://www.olympedia.org/statistics/medal/country), reflecting their broader sports excellence. Furthermore, this note indicates that performance in Masters Swimming is related to performance in Olympic competitions, suggesting that several Masters swimmers may be ex-athletes who competed in the Olympic Games or other high-level competitions. In fact, most of the Masters swimmers can be categorized either as “continuers” (who transitioned directly from youth to Masters swimming) or “rekindlers” (who took a break after their early career before returning to swimming) [45]. Additionally, recent bibliometric analyses covering the past several decades have shown that at least two of the “Big Six” nations (namely the USA and Great Britain) have been among the most prolific contributors to scientific literature on swimming and sports science more broadly [46,47]. This underscores the important role that access to, and the application of, scientific knowledge plays in achieving competitive success. In the context of Masters swimming, the integration of evidence-based training methods, performance analysis, and sport-specific research likely contributes to the sustained excellence observed in these countries.

**Fig 2:**
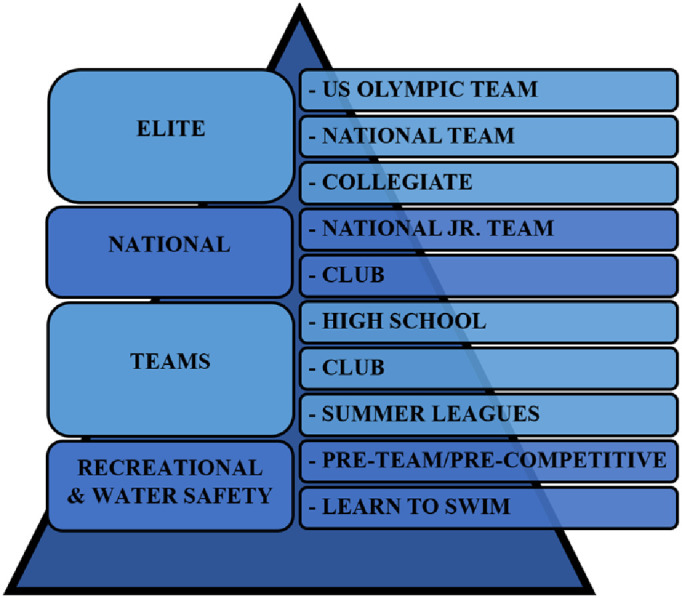
The USA Swimming pyramid according to USA Swimming. a) https://www.usaswimming.org/about-usas/organization/overview.

Correspondingly, the architecture of U.S. competitive swimming is built like a pyramid, with elite swimmers on top and under the umbrella of USA Swimming Fig 2 (https://www.usaswimming.org/). The robust and extensive university swimming system serves as its massive core, allowing younger athletes to dedicate multiple years to training after universities recruit them out of high school for national competitions [48].

Nowadays, performance and professionalism are at such a high level that amateur athletes have little to no chance of winning medals and finals at international events [49]. Amateurs have been displaced by professionals who have devoted their lives to their dreams and accomplishments [50]. Competition results reflect this change, as the level of performance has continually developed [51]. So, where do these professional and elite swimmers end up when they retire? Masters Swimming seems to be a definite option [52].

Thus, it was shown that Masters swimmers improved particularly in older age groups, gradually closing the gap in athletic performance between younger and older competitors through increased participation in more significant competitions, improved nutrition, dryland training, and coaching [53]. The two main factors discussed for this are the participation of former and current elite athletes and the successful efforts of the national associations [52].

For the United States, U.S. Masters Swimming (USMS) provides a wide range of resources for a broad mass of swimmers, including a calendar of events, assistance in finding clubs, articles, and videos to enhance performance, and a vast library of workouts for various training styles (http://www.usms.org/). U.S. results at the World Aquatics Championships directly reflect the professionalism and interconnectedness of USMS.

Similar infrastructures exist in other countries with a shared interest in promoting Masters Swimming (www.worldaquatics.com/members/national-federations?region=all& country=), including the rest of the “Big Six”: Germany (www.dsv.de/masterssport/), Great Britain (www.swimming.org/masters/), Russia (www.swimmasters.ru/), Italy (www.federnuoto.it/home/master/), and Brazil (www.cbda.org.br/natacao-master). By actively promoting Masters Swimming and enhancing swimming infrastructure, these countries ensure that athletes have high access to swimming pools, which directly contributes to the number of workouts and the swimmers’ performance [24].

This study allowed us to analyze a broad spectrum of Masters swimmers from different countries and highlight important information about the origins of the most frequent Masters swimmers among the top-ten performers in all four disciplines. As hypothesized, U.S. swimmers are disproportionately represented among the top-ten finishers in Masters swimming much like their outstanding and consistent success at the Olympic Games, although German female swimmers accounted for the most top-ten finishers in all breaststroke and butterfly distances, while U.S. women ranked second in terms of numbers. However, this research has several limitations related to its design: (i) this is only a time-based research analysis; (ii) no biomechanical or physiological determinants were analyzed; (iii) no information about the training conditions was considered; (iv) it is possible that the same athlete who performed among the top-ten over multiple years had their performance included multiple times in the analysis, which could affect the independence of the observations but cannot be excluded due to the format of the database; (v) the age group analysis is a retrospective cross-sectional observation; therefore, the decline in performance in different age groups should be interpreted with caution as it involves distinct athletes; (vi) no data were present about the number of former or current high-level professional and elite swimmers among the participants; (vii) the inherent selection bias caused by focusing on the top-ten annual times, which may not reflect the broader participation trends of the general Masters swimming population; (viii) the performance analysis was conducted on aggregate data without adjusting for age distribution within the top-ten participants. While age is a primary determinant of performance in Masters swimming, this study focused on global national success; however, the lack of age-standardization should be considered a limitation as it may introduce confounding effects if a nation’s elite participants are concentrated in specific age categories. (ix) Non-parametric tests were used instead of multilevel models due to the unavailability of unique swimmer identifiers. The absence of unique swimmer identifiers in the public World Aquatics Masters archive precludes a hierarchical mixed-effects analysis with performances nested within swimmers. Such a model would more rigorously partition variance across years, events, and individual athletes, and represents an important extension once linked individual-level data become available. Relatedly, all findings should be interpreted strictly at the national level: the cumulative frequency metric quantifies the breadth and longevity of national representation in elite finals and does not support inferences about the average ability of any individual swimmer of a given nationality.

Upcoming studies should compare training variables (such as volume, intensity, and frequency), competition history, and coaches’ expertise. A comparison of biomechanical and physiological variables would provide a better understanding of why some regions develop faster swimmers. Finally, data about the intersection between high-level professional and elite athletes on one side and recreational and amateur swimmers on the other should be observed.

## Conclusions

This study confirmed the numerical superiority of swimmers from the USA among the top-ten performers at the World Aquatics Championships held between 1986 and 2024. In summary, especially U.S. male swimmers were the most frequently represented among the top-ten in every discipline and at nearly all distances. At the same time, the USA had the most frequent Masters swimmers among the top-ten performers, also predominantly in the backstroke and freestyle disciplines during female competitions. The Number of German female swimmers, on the other hand, made up the majority in almost all breaststroke and butterfly events, with U.S. female athletes close behind in both. Additionally, Brazilian men excelled in the 50 m backstroke, while Russian men led the 50 m breaststroke, by having the most frequently represented Masters swimmers in the top-ten. The “Big Six” among the top-ten performers, across all years and disciplines, were the USA, Germany, Great Britain, Russia, Italy and Brazil.

The results of the study can be helpful, primarily in identifying the countries from which the top-performing Masters swimmers originate and secondarily in investigating the specifics of the best-performing Masters swimming nations. The data should serve as a basis for future studies to identify the contributing factors to this success, which can facilitate the development of policies for improving more effective training programs and coaching strategies. Coaches and trainers could optimize training goals by improving training patterns in line with those of the most successful nations in Masters swimming.

## Supporting information

S1 Table FINA World Masters Championships/World Aquatics Championships 1986–2024. According to www.worldaquatics.com/masters/archives/masters-archive.
(TIF)
