# Supplementary material for: Performance patterns and records in the world aquatics masters championships: Where do the most frequently represented nations among the top-ten masters swimmers come from?
Source: PLoS One. 2026 Jun 30;21(6):e0352386. doi: 10.1371/journal.pone.0352386 (PMC13318016; doi:10.1371/journal.pone.0352386)
Supplement: Attachment — Submitted filename: Comments and Suggestions for Plosone_25_8_2025.pdf [file pone.0352386.s002.pdf]

Comments and Suggestions for Authors (#Manuscript PONE-D-25-29285)

Thank you for the opportunity to review the paper titled “Men from USA and Women from Germany Are the Fastest Masters Swimmers in the World.” This study addresses a pertinent subject with an extensive championship dataset that encompasses several decades, strokes, distances, and age demographics. Its advantages encompass extensive coverage and a well-defined descriptive objective. Nonetheless, the framework and methodologies necessitate considerable enhancement prior to substantiating the claims. Specifically, the term "fastest" requires a clear, predetermined definition; comparisons must consider pool length, event variety, age distribution, and participation density; repeated measures at the athlete level and country clustering necessitate multilevel modeling; and nationality coding alongside geopolitical changes demands sensitivity analyses to prevent ecological fallacy. Visualizations ought to depict effect sizes accompanied by uncertainty instead of league tables. By formulating clearer research goals, implementing an analysis plan centered on adjusted country effects (incorporating multiple-testing control), and providing open data/code, the work might offer a valuable descriptive addition. I trust the comprehensive feedback will help the authors in enhancing the manuscript.

I appreciate the opportunity to evaluate this work. The research topic is pertinent, and the analysis of an extensive dataset of Masters swimming performances constitutes a significant contribution to the area. Although the work shows promise, I contend that minor modification is essential to rectify some critical aspects that will augment the manuscript's academic rigor and refinement. My determination for one minor change is predicated on the subsequent overarching observations:

General comments:

1.Title Refinement and Equilibrium: The existing title asserts a robust, conclusive statement that is inadequately substantiated by the intricate facts presented in the abstract. The results indicate that several countries excel in particular strokes and lengths, hence complicating the assertion of a solitary "fastest" designation. I suggest rewording the title to better reflect the data's intricacy and to more accurately correspond with the findings, emphasizing a "preponderance of top-ten finishes" instead of a general assertion of being the "fastest." This will precisely represent the study's scope and maintain academic integrity. This is a suggestion, but it depends on the individual opinion of the authors.

2.The title and several sections of the discussion disproportionately highlight nationality as the predominant determinant in success in Masters swimming. Although this conclusion is intriguing, the manuscript's commentary appropriately highlights other significant elements such as a nation's swimming infrastructure, historical legacies, and particular training methods. I propose a slight modification to the title and discussion to guarantee a balanced viewpoint, recognizing that nationality serves as a proxy for a multitude of intricate, underlying issues. This will offer a more comprehensive and compelling analysis for the reader.

Specific comments:

Abstract:

1.Lines 64–72: The methods should explicitly mention long-course (50 m), define the unit of analysis (top-10 entries per year by distance, sex, and age), address the treatment of duplicate swimmer-year entries, and clearly identify the statistical tests employed.

2. Lines 73 to 88: Results include ordinal terminology (“first/second place”); delineate the measure (counts vs mean time) and present magnitudes (e.g., proportion of top-10 positions). Ensure that the conclusion "fastest... originated" accurately represents that measure, rather than simply the frequency of occurrences.

Introduction:

1. Lines 97–110: The introduction contrasts : Masters and top athletes but appears promotional; refine to eliminate subjective language (“comes as no surprise”), incorporate citations for inclusivity, networking, and coached workout assertions, and more precisely characterize the target adult population.

2. Lines 112–123 provide useful historical context; nevertheless, the co-location of events since 2015 and the 14 age groups appear to be at a methodological level—consider relocating or compressing this information. Additionally, validate the "growing interest" by presenting multi-year participation data, rather than solely using the 2017 peak.

3. Lines 146–170: The objective is evident, although "origin" requires operationalization (nationality, federation license, birthplace, residence). Avoid suggesting causal "reasons" unless validated; distinguish descriptive objectives from hypotheses. Ensure that the word "long course (50 m)" is precise and consistent.

Methods

1. Lines 205–209 (Pandemic gap & recoding): clarify the cancellations of championships (2020–2022) and record the recoding of “NIA” to “RUS.” Present justification, sensitivity analyses with and without recoding, and implications for national counts. Consider preserving both codes in supplemental data for transparency.

Statistical Analysis

1. Lines 219–222 Limiting analyses to the top-10 times introduces selection bias and obscures the analytical unit. Kindly specify if the observations represent swimmer-years or country-year aggregates, explain the methodology for addressing recurrent appearances, and incorporate sensitivity analyses utilizing alternative cutoffs (e.g., top-5, top-20, percentile thresholds).

2. Lines 221–228: The aggregate of nationalities (top five against "Others") may be diverse and based on data. Kindly provide a priori justification for this rule, provide the group sizes for each comparison, evaluate the impacts of imbalance, and explore alternatives such as hierarchical or mixed models, or the exclusion of "Others" in sensitivity studies. This is a suggestion, but it depends on the individual opinion of the authors.

3. Lines 229–234: Normality/variance tests are noted, but Kruskal–Wallis assumes independent samples and does not adjust for covariates (age-group, year). Please verify independence, report effect sizes (e.g., ε^2), control family-wise/FDR across many tests, and consider stratified or mixed-effects models. Please verify !

Results

1. Lines 236–247: Please clarify whether the participation percentages are derived from the complete dataset or the previously mentioned “top-10” subset. Clearly specify the unit of analysis (swimmer-year versus country-year) and present the relevant denominators to ensure transparency and reproducibility.

2. Lines 260–263: The description of sex distribution would be enhanced by the standardization or stratification by age group. Variations in age distribution by sex could obscure participation trends; consider age-adjusted analyses or direct standardization to enhance clarity across events.

3. Lines 264–273: Consolidating all nations outside the top five into a "Others" category results in a heterogeneous comparator, potentially skewing post-hoc comparisons. Kindly include the group sizes for each cell, justify this aggregate in advance, and include sensitivity analyses utilizing other groupings or hierarchical models.

4.Table 4-10: Post-hoc comparisons such as “BRA vs JPN” lack the inclusion of corrected p-values and effect sizes. Please verify, (if it possible) to present the adjusted p-values (Bonferroni/FDR), indicate the direction of differences, and provide an effect size for the Kruskal–Wallis test (e.g., ε^2 or η^2H) along with confidence intervals for each contrast.

5. Correct a Table 2 discrepancy and address outliers. The text indicates that the trends for the 100 m and 200 m events are analogous and directs readers to Table 2, which is entitled “Fastest 50 m Mean Race Times by Stroke.” Either (a) rename Table 2 to specify that it pertains just to 50 m and provide corresponding summary tables for 100 m and 200 m, or (b) rephrase the wording. Table 2 also presents implausible or erroneous maxima (e.g., 1903.00 s for the 50 m butterfly).

6. Numeric formatting : Values like “12’876” with df=5 and p=0.025 seem to employ a thousands apostrophe in place of a decimal (presumably the value of 12,876). Kindly standardize decimal notation and validate all test statistics.

7. In Table 4 (male backstroke 50 m), Japan's mean time of 27.32 seconds surpasses Brazil's 28.29 seconds; still, Brazil is rated first while Japan is ranked fifth. Eliminate the “Ranking” column or specify its criteria (e.g., based on frequency of top-10 appearances rather than speed) and organize the tables according to the chosen metric. The current rank order violates the means and confounds readers.

8. Propose a singular comprehensive figure accompanied by more concise tables. Replace four nationality tables with a singular heatmap (country × stroke × distance × sex; cells represent standardized mean time or proportion in annual top-10). Retain Table 1 (participation) and a refined Table 2 (summary of

50/100/200 m), and transfer the comprehensive post-hoc tables to the Supplementary section. This will reduce the main-text tables from 10 to approximately 3, enhancing scanability. This is a suggestion, but it depends on the individual opinion of the authors.

9. Please standardize "50 m" against "50m," align decimal points, and ensure consistent formatting of "±" throughout all tables. Although minor, it enhances clarity when you condense and renumber.

Discussion:

1. Lines 394 – 403: The Discussion starts by repeating the study's scope, methodologies, and the preceding hypothesis of Olympic dominance. Kindly summarize this recap and shift to interpretation and clearly articulate how the results assess the hypothesis using statistical analysis.

2. Lines 405–419: The "main findings" list collects top-ten appearances but fails to correlate them with your inferential tests. Please consider present comparable effect sizes and corrected p-values, and specify if “most top-ten entries” serves as an equivalent for “fastest.”

3. Lines 454–479: Genetic, meteorological, and open-water factors are inferred from running/open-water literature to analyze pool Masters results. Clarify the external validity about long-course pool performance, avoid genetic determinism, and emphasize swimming-specific factors (starts, turns, lanes availability, and coaching culture) with precise citations. This is a suggestion, but it depends on the individual opinion of the authors.

4. Lines 481–496: Socioeconomic comparisons contrast U.S. and China in terms of population and income; nevertheless, the framework for China's Masters engagement is not delineated. Align comparators to Masters-specific contexts and utilize rate-based metrics instead of absolute counts.

5. Lines 524–536 & 551–556: The USA Swimming "pyramid" (Table 11) and the USMS narrative appear to be promotional in nature. Establish empirical connections to Masters outcomes (e.g., transition rates from NCAA/elite to Masters; club representation by state) or refine to eliminate unfounded causal inferences.

6. Lines 570–583: The limitations are useful; please consider incorporate sensitivity analysis for (a) selection bias due to annual top-ten cutoffs, (b) repeated-measures dependence (same athlete over years), and (c) alterations in country-level coding; consider robustness tests for unique athletes and per-country weighting. This is a suggestion, but it depends on the individual opinion of the authors.

Conclusion:

1.Lines 596–602: The implication statements are general and conclude with rhetoric (“Swimming was, is, and will remain…”). Substitute with specific, actionable insights that correspond to your constraints (selection, repeated measures), and maintain uniform nomenclature (prefer "World Aquatics" over the inconsistent use of "FINA/World Aquatics"). This is a suggestion, but it depends on the individual opinion of the authors.
