# Supplementary material for: Performance patterns and records in the world aquatics masters championships: Where do the most frequently represented nations among the top-ten masters swimmers come from?
Source: PLoS One. 2026 Jun 30;21(6):e0352386. doi: 10.1371/journal.pone.0352386 (PMC13318016; doi:10.1371/journal.pone.0352386)
Supplement: Attachment — Submitted filename: First round Comments_15_March_2026.pdf [file pone.0352386.s004.pdf]

Review Comments to the Author

The manuscript presents a comprehensive dataset of Masters swimming results covering almost four decades. Nonetheless, other technical and interpretive challenges remain, especially concerning selection bias (top-10 data), repeated measures, insufficient correction for participation rates, and possible ecological fallacies in the interpretation of nationality differences. Addressing these issues would significantly enhance the scientific rigor and clarity of the work.

General comments

1. The term "performance trends" implies a thorough chronological examination of performance development, although the manuscript mostly presents descriptive comparisons of national representation within the top ten race results. Please verify !
2. The concept of "most successful Masters swimmers" is not clearly defined. The title implies a clear and objective metric of success, yet the study operationalizes success primarily through frequency of top-ten appearances and mean race times, which is not explicitly conveyed.
3. The title does not reflect key methodological characteristics of the study. Essential aspects such as the focus on Masters swimming competitions and the use of top-ten performance records are absent, which reduces transparency regarding the analytical approach.

Specific comments

Abstract

1.The title and abstract refer to "performance trends," yet the analysis appears largely descriptive regarding national representation among top-10 performances. Does the study actually quantify temporal performance trends (e.g., changes in race times across years)?
2. The abstract refers to the "most successful Masters swimmers." How is success operationally defined—mean race time, frequency of top-ten appearances, or both?
3. The methods section states that top-10 entries were used as the unit of analysis. Does this approach introduce selection bias by excluding the broader performance distribution of Masters swimmers?

Introduction

1. The introduction suggests that nationality may influence performance through genetic, socioeconomic, or environmental factors. Are these hypotheses explicitly tested in the study design?
2. The hypothesis that U.S. athletes would dominate Masters swimming appears to be based primarily on elite swimming history. Is this assumption justified given the different demographic structure of Masters sport?

Materials and Methods

1. The study considers each top-10 performance entry as a unique observation, but individual swimmers may appear many times across different years or races. In what manner was repeated-measures bias addressed?
2. The unit of analysis is defined as "swimmer-year entries." Does this approach risk inflating the influence of frequently competing athletes?
3. The analysis aggregates all age groups within each sex. Could differences in age distribution across countries confound the results?
4. The dataset includes results from 1986–2024, yet competitions did not occur between 2020–2022 due to the pandemic. How might this interruption affect trend interpretation?
5. Russian athletes competing under the designation "NIA" were recoded as "RUS." Was a sensitivity analysis conducted to evaluate how this recoding might influence nationality comparisons?
6. The Kruskal–Wallis test was used to compare nationalities. Given the hierarchical data structure (years, events, swimmers, countries), would mixed-effects or multilevel models provide a more appropriate analytical framework?

Results

1.The results emphasize country rankings based on top-ten appearances, yet mean race times are also reported. Which metric is used to define "success"?
2. Table 2 reports extreme maximum values (e.g., >1900 s for a 50 m butterfly event). Were data-cleaning procedures performed to detect potential recording errors?
3. Many comparisons across strokes, distances, and countries are reported. How was multiple testing controlled beyond Bonferroni correction?
4. The tables focus on descriptive comparisons. Would graphical visualization (e.g., heatmaps or trend plots) improve interpretability of nationality patterns?

Discussion and Conclusion

1.The discussion interprets nationality differences as reflecting training systems or sport infrastructure. However, these variables were not directly measured. Are such interpretations speculative?
2. The manuscript suggests that U.S. success may be due to structured swimming systems. Could this pattern also be explained by larger participation pools rather than performance advantages?

3. The discussion occasionally conflates “fastest swimmers” with “most frequent top-ten appearances.” Are these two constructs equivalent?
4. Does the discussion adequately acknowledge the ecological fallacy risk, where group-level patterns are interpreted as individual-level determinants?
5. The findings are described as identifying geographic patterns of excellence. However, does the study actually establish causal mechanisms behind these patterns?
6. The conclusion suggests implications for future research and training systems, yet the current analysis is descriptive. How should these findings be translated into practical recommendations?
7. Would a more cautious conclusion emphasizing descriptive nationality patterns rather than national superiority better reflect the limitations of the study?
