# Supplementary material for: Performance patterns and records in the world aquatics masters championships: Where do the most frequently represented nations among the top-ten masters swimmers come from?
Source: PLoS One. 2026 Jun 30;21(6):e0352386. doi: 10.1371/journal.pone.0352386 (PMC13318016; doi:10.1371/journal.pone.0352386)
Supplement: Attachment — Submitted filename: PONE-D-25-29285_Revision 2_Responses.docx [file pone.0352386.s006.docx]

**Responses to the Reviewer**

*Reviewer: The manuscript presents a comprehensive dataset of Masters swimming results covering almost four decades. Nonetheless, other technical and interpretive challenges remain, especially concerning selection bias (top-10 data), repeated measures, insufficient correction for participation rates, and possible ecological fallacies in the interpretation of nationality differences. Addressing these issues would significantly enhance the scientific rigor and clarity of the work.* **Response:** We thank the expert reviewer for the thorough and constructive evaluation. Below we address each comment point by point. All changes in the revised manuscript are indicated in tracked changes.

***General comments***

*Reviewer: 1. The term "performance trends" implies a thorough chronological examination of performance development, although the manuscript mostly presents descriptive comparisons of national representation within the top ten race results. Please verify!* **Response:** We would like to thank the expert reviewer for this recommendation. We have revised the title and abstract accordingly, replacing “performance trends” with “performance patterns” to better reflect the descriptive comparison of the design.

*Reviewer: 2. The concept of “most successful Masters swimmers” is not clearly defined. The title implies a clear and objective metric of success, yet the study operationalizes success primarily through frequency of top-ten appearances and mean race times, which is not explicitly conveyed.*

**Response:** We thank the expert reviewer for this supportive advice. We have amended the title accordingly, replacing “the most successful Masters swimmers” with the “most frequently represented nations among the top-10 Masters swimmers”.

*Reviewer: 3. The title does not reflect key methodological characteristics of the study. Essential aspects such as the focus on Masters swimming competitions and the use of top-ten performance records are absent, which reduces transparency regarding the analytical approach.*

**Response:** We thank the reviewer for this notice. By adjusting the title, we have highlighted now the focus on Masters swimming competitions and the use of top-10 performance records in our study.

***Specific comments***

***Abstract***

*Reviewer: 1. The title and abstract refer to “performance trends,” yet the analysis appears largely descriptive regarding national representation among top-10 performances. Does the study actually quantify temporal performance trends (e.g., changes in race times across years)?*

**Response:** We thank the expert reviewer and agree that the term “performance trends” overstates the temporal component of the analysis. The study characterizes national representation patterns among top-ten performers rather than modeling changes in race times over time. We have revised the title and abstract accordingly, replacing “performance trends” with “performance patterns” to better reflect the descriptive, cross-sectional nature of the design.

*Reviewer: 2. The abstract refers to the “most successful Masters swimmers.” How is success operationally defined—mean race time, frequency of top-ten appearances, or both?*

**Response:** We appreciate this important comment. Success is operationally defined as the frequency of a nation’s appearances in the annual top-ten fastest times for each stroke, distance, and sex. Mean race times are reported as complementary descriptive statistics but do not define the ranking criterion. We have revised the abstract and the opening of the Results section to state this explicitly.

*Reviewer: 3. The methods section states that top-10 entries were used as the unit of analysis. Does this approach introduce selection bias by excluding the broader performance distribution of Masters swimmers?*

**Response:** We thank the expert reviewer for pointing this out. Yes, this is an inherent feature of the design. The study intentionally focuses on the elite tier of Masters performance to characterize national dominance patterns, not to describe the broader competitive population. This selection is acknowledged as a limitation in the Discussion (item vii) and we have added a clarifying sentence in the Methods section.

***Introduction***

*Reviewer: 1. The introduction suggests that nationality may influence performance through genetic, socioeconomic, or environmental factors. Are these hypotheses explicitly tested in the study design?* **Response:** We thank the expert reviewer for his valuable observation. Although genetic, socioeconomic, or environmental factors are discussed, they are not explicitly examined in the study design. The primary aim of our study is to identify the nations from which the top-performing Masters swimmers originate most frequently and to lay the basis for future studies to investigate and identify the factors contributing to this success in greater detail.

*Reviewer: The hypothesis that U.S. athletes would dominate Masters swimming appears to be based primarily on elite swimming history. Is this assumption justified given the different demographic structure of Masters sport?*	**Response:** We acknowledge the expert reviewer’s concern regarding our hypothesis, which is partly based on elite swimming; however, there have been no comprehensive studies on the performance patterns of Masters swimmers. Given the potential overlap and a “fluidity” between both, we have therefore based our hypothesis on elite swimming.

***Materials and Methods***

*Reviewer: 1. The study considers each top-10 performance entry as a unique observation, but individual swimmers may appear many times across different years or races. In what manner was repeated-measures bias addressed?*

**Response:** We appreciate the expert reviewer’s comment on repeated-measures bias. Due to the structure of the publicly available World Aquatics database, consistent unique swimmer identifiers are not available, which precluded formal repeated-measures modeling. Each entry is treated as an independent performance record within its competition context. We recognize that this may inflate the apparent contribution of prolific individual competitors and have expanded the limitations section accordingly.

*Reviewer: 2. The unit of analysis is defined as “swimmer-year entries.” Does this approach risk inflating the influence of frequently competing athletes?*

**Response:** We appreciate the expert reviewer’s concern. This concern is related to the previous point and we acknowledge it. An athlete appearing in the top ten across multiple years contributes multiple entries, which inflates their nation’s representation count. Without reliable unique-identifier linkage, this effect cannot be fully quantified. We have noted this as a limitation and suggest that future studies with linked athlete-level data assess the robustness of these findings to individual-level clustering.

*Reviewer: 3. The analysis aggregates all age groups within each sex. Could differences in age distribution across countries confound the results?*

**Response:** We thank the expert reviewer for this valid concern. If a nation’s top-ten appearances are concentrated in younger (and therefore faster) age groups, its aggregate mean time would appear faster without reflecting superior performance at comparable ages. A full age-stratified analysis across all combinations (4 strokes × 3–5 distances × 2 sexes × 12 age groups × 6 nationality categories) was beyond the scope of this study, but we have acknowledged this as a limitation and recommend age-standardized comparisons in future work.

*Reviewer: 4. The dataset includes results from 1986–2024, yet competitions did not occur between 2020–2022 due to the pandemic. How might this interruption affect trend interpretation?*

**Response:** We appreciate the opportunity to clarify this point. The three-year gap reduces the total number of observations but does not create a directional bias, since our analysis is based on cumulative frequency counts per nationality rather than time-series modeling. We have added a note in the Methods section acknowledging the interruption and clarifying that the 2020–2022 period was excluded due to the absence of competitions.

*Reviewer: 5. Russian athletes competing under the designation “NIA” were recoded as “RUS.” Was a sensitivity analysis conducted to evaluate how this recoding might influence nationality comparisons?*

**Response:** We appreciate the expert reviewer’s attention. The recoding applies exclusively to the 2023 and 2024 editions (2 of 20 championships), restoring continuity with the preceding 34 years of consistent RUS coding. Given the small proportion of affected entries relative to the full dataset, the impact on cumulative nationality rankings is negligible. We have added a note in the Methods clarifying the rationale and scope of this recoding decision.

*Reviewer: 6. The Kruskal–Wallis test was used to compare nationalities. Given the hierarchical data structure (years, events, swimmers, countries), would mixed-effects or multilevel models provide a more appropriate analytical framework?*

**Response:** We thank the expert reviewer for this meaningful comment and agree that the data have a hierarchical structure and that mixed-effects models would in principle be more appropriate. However, two constraints limited this approach: (a) unique swimmer identifiers were not reliably available, precluding specification of a swimmer-level random effect; and (b) the primary research question concerned frequency of national representation rather than variance decomposition of race times. We have acknowledged this as a methodological limitation and recommend multilevel approaches for future studies with richer individual-level data.

***Results***

*Reviewer: 1. The results emphasize country rankings based on top-ten appearances, yet mean race times are also reported. Which metric is used to define “success”?*

**Response:** We thank the expert reviewer for pointing this out. As clarified in our response to Abstract comment 2, the primary metric is frequency of top-ten appearances per nationality. Mean race times are reported descriptively to illustrate the performance level within each national group. We have revised the opening of the Results section to make this distinction clear.

*Reviewer: 2. Table 2 reports extreme maximum values (e.g., >1900 s for a 50 m butterfly event). Were data-cleaning procedures performed to detect potential recording errors?*

**Response:** We thank the expert reviewer for identifying this. The value of 1903 s for a 50 m butterfly event is clearly a data entry error in the original World Aquatics archive and does not represent a valid performance. We have flagged this entry in the revised Table 2 with a footnote indicating it as a recording artifact. Its exclusion does not alter the mean values, standard deviations, or statistical comparisons reported, as confirmed by inspection.

*Reviewer: 3. Many comparisons across strokes, distances, and countries are reported. How was multiple testing controlled beyond Bonferroni correction?*

**Response:** We thank the expert reviewer for the opportunity to clarify this point. Bonferroni correction was applied to all pairwise post-hoc comparisons within each Kruskal–Wallis test. No additional correction was applied across the separate omnibus tests (one per stroke/distance/sex combination), as the study is exploratory and descriptive in nature. Applying a further family-wise correction across all tests would be excessively conservative and would obscure meaningful patterns. We have added a sentence in the Methods noting this decision and recommending that the reported p-values be interpreted with appropriate caution.

*Reviewer: 4. The tables focus on descriptive comparisons. Would graphical visualization (e.g., heatmaps or trend plots) improve interpretability of nationality patterns?*

**Response:** We appreciate the expert reviewer’s suggestion. The current tabular format was chosen to preserve the full granularity of the data across strokes, distances, sexes, and nationalities. Synthetic visualizations such as heatmaps would necessarily aggregate across some of these dimensions, potentially obscuring the stroke- and distance-specific patterns that are central to our findings.

***Discussion and Conclusion***

*Reviewer: 1. The discussion interprets nationality differences as reflecting training systems or sport infrastructure. However, these variables were not directly measured. Are such interpretations speculative?* **Response:** We thank the expert reviewer for the meaningful criticism. In our discussion, we explained how professionally and seamlessly swimming appears to be organized in the USA. This could well be a key factor in the success and the possible interconnection between U.S. elite and Masters swimmers, and merits closer examination in future studies.

*Reviewer: 2. The manuscript suggests that U.S. success may be due to structured swimming systems. Could this pattern also be explained by larger participation pools rather than performance advantages?* **Response:** We thank the expert reviewer for the opportunity to clarify this point. have discussed that population size and prosperity are among the most important socioeconomic factors for success. In this context, by comparing the population sizes of China and the USA, as well as the success of athletes from countries with significantly smaller populations, we have shown that whilst a larger pool of potential athletes appears advantageous, it may only be decisive for the development of successful athletes in certain cases.

*Reviewer: 3. The discussion occasionally conflates “fastest swimmers” with “most frequent top-ten appearances”. Are these two constructs equivalent?* **Response:** We thank the expert reviewer for this valued question and the opportunity to improve the manuscript by correcting the “fastest swimmers” to “the most frequent Masters swimmers among the top-ten performers” throughout the manuscript.

*Reviewer: 4. Does the discussion adequately acknowledge the ecological fallacy risk, where group-level patterns are interpreted as individual-level determinants?* **Response:** We thank the expert reviewer for this critical comment. Since we analyzed multiple individual athletes' performance patterns and records over time to identify the most frequently represented nations among the top-ten Masters swimmers, there should be no conflict with an ecological fallacy risk.

*Reviewer: 5. The findings are described as identifying geographic patterns of excellence. However, does the study actually establish causal mechanisms behind these patterns?* **Response:** We thank the expert reviewer for this constructive question. Although environmental factors are discussed, such as geographical patterns, they are not operationalized. According to the literature, there is a clear link between geographical and climatic conditions and the success of nations—albeit in open-water swimming.

*Reviewer: 6. The conclusion suggests implications for future research and training systems, yet the current analysis is descriptive. How should these findings be translated into practical recommendations?* **Response:** We thank the expert reviewer for this attentive remark. Upcoming studies should compare training variables (such as volume, intensity, and frequency), competition history, and coaches’ expertise. A comparison of biomechanical and physiological variables would provide a better understanding of why some regions develop faster swimmers. Coaches and trainers could optimize training goals by improving training patterns in line with those of the most successful nations in Masters swimming.

*Reviewer: 7. Would a more cautious conclusion emphasizing descriptive nationality patterns rather than national superiority better reflect the limitations of the study?* **Response:** We were very happy to accept the expert reviewer’s valuable suggestions and have tried to tone down the summary, by more cautious conclusions und by describing more nationality patterns.

We trust that these revisions adequately address the reviewer’s concerns and improve the clarity and rigor of the manuscript.

**All comments made by Reviewer 4 appear to refer to a different manuscript and have therefore not been taken into account or addressed.**
