# Supplementary material for: Performance patterns and records in the world aquatics masters championships: Where do the most frequently represented nations among the top-ten masters swimmers come from?
Source: PLoS One. 2026 Jun 30;21(6):e0352386. doi: 10.1371/journal.pone.0352386 (PMC13318016; doi:10.1371/journal.pone.0352386)
Supplement: Peer review history

# Peer review: Decision Letter 0

**DOI:** 10.1371/journal.pone.0352386.r001

**Authors:** Rodrigo Zacca

23 Sep 2025

PONE-D-25-29285 Men from USA and Women from Germany Are the fastest Masters Swimmers in the World PLOS ONE

Dear Dr. Knechtle,

Thank you for submitting your manuscript to PLOS ONE. After careful consideration, we feel that it has merit but does not fully meet PLOS ONE’s publication criteria as it currently stands (**MAJOR REVIEW)**. Therefore, **we invite you to submit a revised version of the manuscript** that addresses the points raised during the review process.

Please submit your revised manuscript by Nov 07 2025 11:59PM. If you will need more time than this to complete your revisions, please reply to this message or contact the journal office at plosone@plos.org. When you're ready to submit your revision, log on to https://www.editorialmanager.com/pone/ and select the 'Submissions Needing Revision' folder to locate your manuscript file.

Please include the following items when submitting your revised manuscript:

- A rebuttal letter that responds to each point raised by the academic editor and reviewer(s). You should upload this letter as a separate file labeled 'Response to Reviewers'.
- A marked-up copy of your manuscript that highlights changes made to the original version. You should upload this as a separate file labeled 'Revised Manuscript with Track Changes'.
- An unmarked version of your revised paper without tracked changes. You should upload this as a separate file labeled 'Manuscript'.

If you would like to make changes to your financial disclosure, please include your updated statement in your cover letter. Guidelines for resubmitting your figure files are available below the reviewer comments at the end of this letter.

If applicable, we recommend that you deposit your laboratory protocols in protocols.io to enhance the reproducibility of your results. Protocols.io assigns your protocol its own identifier (DOI) so that it can be cited independently in the future. For instructions see: https://journals.plos.org/plosone/s/submission-guidelines#loc-laboratory-protocols. Additionally, PLOS ONE offers an option for publishing peer-reviewed Lab Protocol articles, which describe protocols hosted on protocols.io. Read more information on sharing protocols at https://plos.org/protocols?utm_medium=editorial-email&utm_source=authorletters&utm_campaign=protocols.

We look forward to receiving your revised manuscript.

Kind regards,

Rodrigo Zacca, Ph.D

Academic Editor

PLOS ONE

Journal Requirements:

When submitting your revision, we need you to address these additional requirements.

1.Please ensure that your manuscript meets PLOS ONE's style requirements, including those for file naming. The PLOS ONE style templates can be found at

https://journals.plos.org/plosone/s/file?id=wjVg/PLOSOne_formatting_sample_main_body.pdf and

https://journals.plos.org/plosone/s/file?id=ba62/PLOSOne_formatting_sample_title_authors_affiliations.pdf

2. Your ethics statement should only appear in the Methods section of your manuscript. If your ethics statement is written in any section besides the Methods, please delete it from any other section.

3. We notice that your supplementary tables are included in the manuscript file. Please remove them and upload them with the file type 'Supporting Information'. Please ensure that each Supporting Information file has a legend listed in the manuscript after the references list.

4. Please include captions for your Supporting Information files at the end of your manuscript, and update any in-text citations to match accordingly. Please see our Supporting Information guidelines for more information: http://journals.plos.org/plosone/s/supporting-information.

5. We note that there is identifying data in the Supporting Information file “FINA1986-2024 aktuell.xlsx”. Due to the inclusion of these potentially identifying data, we have removed this file from your file inventory. Prior to sharing human research participant data, authors should consult with an ethics committee to ensure data are shared in accordance with participant consent and all applicable local laws.

Data sharing should never compromise participant privacy. It is therefore not appropriate to publicly share personally identifiable data on human research participants. The following are examples of data that should not be shared:

-Name, initials, physical address

-Ages more specific than whole numbers

-Internet protocol (IP) address

-Specific dates (birth dates, death dates, examination dates, etc.)

-Contact information such as phone number or email address

-Location data

-ID numbers that seem specific (long numbers, include initials, titled “Hospital ID”) rather than random (small numbers in numerical order)

Data that are not directly identifying may also be inappropriate to share, as in combination they can become identifying. For example, data collected from a small group of participants, vulnerable populations, or private groups should not be shared if they involve indirect identifiers (such as sex, ethnicity, location, etc.) that may risk the identification of study participants.

Additional guidance on preparing raw data for publication can be found in our Data Policy (https://journals.plos.org/plosone/s/data-availability#loc-human-research-participant-data-and-other-sensitive-data) and in the following article: http://www.bmj.com/content/340/bmj.c181.long.

Please remove or anonymize all personal information (<specific identifying information in file to be removed>), ensure that the data shared are in accordance with participant consent, and re-upload a fully anonymized data set. Please note that spreadsheet columns with personal information must be removed and not hidden as all hidden columns will appear in the published file.

6. If the reviewer comments include a recommendation to cite specific previously published works, please review and evaluate these publications to determine whether they are relevant and should be cited. There is no requirement to cite these works unless the editor has indicated otherwise.

Additional Editor Comments:

Dear Authors,

Four experienced reviewers with expertise in swimming have devoted considerable time and effort to evaluate your manuscript. Overall, the decision was heterogeneous.

In this regard, I kindly request that you respond carefully to each of the reviewers’ comments (Major Revision), either by accepting and editing accordingly or by providing well-reasoned rebuttals where appropriate.

Please note that the changes do not need to be tracked; however, they should be highlighted in red within the revised manuscript.

We look forward to receiving your revised submission.

With best regards,

Rodrigo Zacca, Ph.D

Reviewer #1:

The manuscript under review was prepared using public data on world masters swimming results. It is very well-written, presenting comprehensive and interesting data. The discussion is broad and not merely explains the results, but also analyzes what leads to such results. I would only suggest that the rationale for the study be clearer. The introduction, while well-written, does not objectively justify the study's purpose. However, reading the discussion, this becomes clear.

Reviewer #2:

The present study was not original, it only performed limited scope analysis on a pre-existing publicly available data set. Furthermore, the significance of this work is very low. It is not made clear how the findings of this study significantly change anything - for example there is no recommendation for national governing body swimming infrastructure strategy our our identification of understanding what the specific unique circumstances are to foster fast masters swimmers. Instead the study applies post-hoc explanations for the results (e.g., stating that Germany has a well-organized and comprehensive swimming infrastructure through the DSV5, whilst ommiting discussion of other countries that have equally well organised infrastructure).

There are numerous limitations to the statistical analysis that confound the findings and the author's interpretation of them. Not scaling the results for significant factors such as country population size or GDP per capita mean that the author's fail to improve the reader's understanding of how these factors have an impact on success at Master's swimming level. Furthermore, being unable to account for either former professional swimmers or individual swimmers who may compete across multiple years and multiple events is a significant limitation. Without accounting for this all descriptive statistics are likely to be significantly biased and lead to misleading interpretations of which country is best at producing the fastest masters swimmers from purely recreational swimmers.

In summary this submission is being recommended for rejection due to low significance, originality and rigour.

Reviewer #3:

Thank you for the opportunity to review the paper titled “Men from USA and Women from Germany Are the Fastest Masters Swimmers in the World.” This study addresses a pertinent subject with an extensive championship dataset that encompasses several decades, strokes, distances, and age demographics. Its advantages encompass extensive coverage and a well-defined descriptive objective. Nonetheless, the framework and methodologies necessitate considerable enhancement prior to substantiating the claims. Specifically, the term "fastest" requires a clear, predetermined definition; comparisons must consider pool length, event variety, age distribution, and participation density; repeated measures at the athlete level and country clustering necessitate multilevel modeling; and nationality coding alongside geopolitical changes demands sensitivity analyses to prevent ecological fallacy. Visualizations ought to depict effect sizes accompanied by uncertainty instead of league tables. By formulating clearer research goals, implementing an analysis plan centered on adjusted country effects (incorporating multiple-testing control), and providing open data/code, the work might offer a valuable descriptive addition. I trust the comprehensive feedback will help the authors in enhancing the manuscript.

I appreciate the opportunity to evaluate this work. The research topic is pertinent, and the analysis of an extensive dataset of Masters swimming performances constitutes a significant contribution to the area. Although the work shows promise, I contend that minor modification is essential to rectify some critical aspects that will augment the manuscript's academic rigor and refinement. My determination for one minor change is predicated on the subsequent overarching observations:

General comments:

1.Title Refinement and Equilibrium: The existing title asserts a robust, conclusive statement that is inadequately substantiated by the intricate facts presented in the abstract. The results indicate that several countries excel in particular strokes and lengths, hence complicating the assertion of a solitary "fastest" designation. I suggest rewording the title to better reflect the data's intricacy and to more accurately correspond with the findings, emphasizing a "preponderance of top-ten finishes" instead of a general assertion of being the "fastest." This will precisely represent the study's scope and maintain academic integrity. This is a suggestion, but it depends on the individual opinion of the authors.

2.The title and several sections of the discussion disproportionately highlight nationality as the predominant determinant in success in Masters swimming. Although this conclusion is intriguing, the manuscript's commentary appropriately highlights other significant elements such as a nation's swimming infrastructure, historical legacies, and particular training methods. I propose a slight modification to the title and discussion to guarantee a balanced viewpoint, recognizing that nationality serves as a proxy for a multitude of intricate, underlying issues. This will offer a more comprehensive and compelling analysis for the reader.

Specific comments:

Abstract:

1.Lines 64–72: The methods should explicitly mention long-course (50 m), define the unit of analysis (top-10 entries per year by distance, sex, and age), address the treatment of duplicate swimmer-year entries, and clearly identify the statistical tests employed.

2. Lines 73 to 88: Results include ordinal terminology (“first/second place”); delineate the measure (counts vs mean time) and present magnitudes (e.g., proportion of top-10 positions). Ensure that the conclusion "fastest... originated" accurately represents that measure, rather than simply the frequency of occurrences.

Introduction:

1. Lines 97–110: The introduction contrasts : Masters and top athletes but appears promotional; refine to eliminate subjective language (“comes as no surprise”), incorporate citations for inclusivity, networking, and coached workout assertions, and more precisely characterize the target adult population.

2. Lines 112–123 provide useful historical context; nevertheless, the co-location of events since 2015 and the 14 age groups appear to be at a methodological level—consider relocating or compressing this information. Additionally, validate the "growing interest" by presenting multi-year participation data, rather than solely using the 2017 peak.

3. Lines 146–170: The objective is evident, although "origin" requires operationalization (nationality, federation license, birthplace, residence). Avoid suggesting causal "reasons" unless validated; distinguish descriptive objectives from hypotheses. Ensure that the word "long course (50 m)" is precise and consistent.

Methods

1. Lines 205–209 (Pandemic gap & recoding): clarify the cancellations of championships (2020–2022) and record the recoding of “NIA” to “RUS.” Present justification, sensitivity analyses with and without recoding, and implications for national counts. Consider preserving both codes in supplemental data for transparency.

Statistical Analysis

1. Lines 219–222 Limiting analyses to the top-10 times introduces selection bias and obscures the analytical unit. Kindly specify if the observations represent swimmer-years or country-year aggregates, explain the methodology for addressing recurrent appearances, and incorporate sensitivity analyses utilizing alternative cutoffs (e.g., top-5, top-20, percentile thresholds).

2. Lines 221–228: The aggregate of nationalities (top five against "Others") may be diverse and based on data. Kindly provide a priori justification for this rule, provide the group sizes for each comparison, evaluate the impacts of imbalance, and explore alternatives such as hierarchical or mixed models, or the exclusion of "Others" in sensitivity studies. This is a suggestion, but it depends on the individual opinion of the authors.

3. Lines 229–234: Normality/variance tests are noted, but Kruskal–Wallis assumes independent samples and does not adjust for covariates (age-group, year). Please verify independence, report effect sizes (e.g., ε²), control family-wise/FDR across many tests, and consider stratified or mixed-effects models. Please verify !

Results

1. Lines 236–247: Please clarify whether the participation percentages are derived from the complete dataset or the previously mentioned “top-10” subset. Clearly specify the unit of analysis (swimmer-year versus country-year) and present the relevant denominators to ensure transparency and reproducibility.

2. Lines 260–263: The description of sex distribution would be enhanced by the standardization or stratification by age group. Variations in age distribution by sex could obscure participation trends; consider age-adjusted analyses or direct standardization to enhance clarity across events.

3. Lines 264–273: Consolidating all nations outside the top five into a "Others" category results in a heterogeneous comparator, potentially skewing post-hoc comparisons. Kindly include the group sizes for each cell, justify this aggregate in advance, and include sensitivity analyses utilizing other groupings or hierarchical models.

4.Table 4-10: Post-hoc comparisons such as “BRA vs JPN” lack the inclusion of corrected p-values and effect sizes. Please verify, (if it possible) to present the adjusted p-values (Bonferroni/FDR), indicate the direction of differences, and provide an effect size for the Kruskal–Wallis test (e.g., ε² or η²H) along with confidence intervals for each contrast.

5. Correct a Table 2 discrepancy and address outliers. The text indicates that the trends for the 100 m and 200 m events are analogous and directs readers to Table 2, which is entitled “Fastest 50 m Mean Race Times by Stroke.” Either (a) rename Table 2 to specify that it pertains just to 50 m and provide corresponding summary tables for 100 m and 200 m, or (b) rephrase the wording. Table 2 also presents implausible or erroneous maxima (e.g., 1903.00 s for the 50 m butterfly).

6. Numeric formatting : Values like “12’876” with df=5 and p=0.025 seem to employ a thousands apostrophe in place of a decimal (presumably the value of 12,876). Kindly standardize decimal notation and validate all test statistics.

7. In Table 4 (male backstroke 50 m), Japan's mean time of 27.32 seconds surpasses Brazil's 28.29 seconds; still, Brazil is rated first while Japan is ranked fifth. Eliminate the “Ranking” column or specify its criteria (e.g., based on frequency of top-10 appearances rather than speed) and organize the tables according to the chosen metric. The current rank order violates the means and confounds readers.

8. Propose a singular comprehensive figure accompanied by more concise tables. Replace four nationality tables with a singular heatmap (country × stroke × distance × sex; cells represent standardized mean time or proportion in annual top-10). Retain Table 1 (participation) and a refined Table 2 (summary of 50/100/200 m), and transfer the comprehensive post-hoc tables to the Supplementary section. This will reduce the main-text tables from 10 to approximately 3, enhancing scanability. This is a suggestion, but it depends on the individual opinion of the authors.

9. Please standardize "50 m" against "50m," align decimal points, and ensure consistent formatting of "±" throughout all tables. Although minor, it enhances clarity when you condense and renumber.

Discussion:

1. Lines 394 – 403: The Discussion starts by repeating the study's scope, methodologies, and the preceding hypothesis of Olympic dominance. Kindly summarize this recap and shift to interpretation and clearly articulate how the results assess the hypothesis using statistical analysis.

2. Lines 405–419: The "main findings" list collects top-ten appearances but fails to correlate them with your inferential tests. Please consider present comparable effect sizes and corrected p-values, and specify if “most top-ten entries” serves as an equivalent for “fastest.”

3. Lines 454–479: Genetic, meteorological, and open-water factors are inferred from running/open-water literature to analyze pool Masters results. Clarify the external validity about long-course pool performance, avoid genetic determinism, and emphasize swimming-specific factors (starts, turns, lanes availability, and coaching culture) with precise citations. This is a suggestion, but it depends on the individual opinion of the authors.

4. Lines 481–496: Socioeconomic comparisons contrast U.S. and China in terms of population and income; nevertheless, the framework for China's Masters engagement is not delineated. Align comparators to Masters-specific contexts and utilize rate-based metrics instead of absolute counts.

5. Lines 524–536 & 551–556: The USA Swimming "pyramid" (Table 11) and the USMS narrative appear to be promotional in nature. Establish empirical connections to Masters outcomes (e.g., transition rates from NCAA/elite to Masters; club representation by state) or refine to eliminate unfounded causal inferences.

6. Lines 570–583: The limitations are useful; please consider incorporate sensitivity analysis for (a) selection bias due to annual top-ten cutoffs, (b) repeated-measures dependence (same athlete over years), and (c) alterations in country-level coding; consider robustness tests for unique athletes and per-country weighting. This is a suggestion, but it depends on the individual opinion of the authors.

Conclusion:

1.Lines 596–602: The implication statements are general and conclude with rhetoric (“Swimming was, is, and will remain…”). Substitute with specific, actionable insights that correspond to your constraints (selection, repeated measures), and maintain uniform nomenclature (prefer "World Aquatics" over the inconsistent use of "FINA/World Aquatics"). This is a suggestion, but it depends on the individual opinion of the authors.

Reviewer #4:

Dear Authors,

I want to express my gratitude for the opportunity to review this manuscript.

It's an interesting and important topic in the field of sport, particularly in swimming.

At this stage, the document requires improvements, below with line indication:

88 - Please revise the upper and lowercase criteria.

111/124/131 - Please revise the line spacing criteria. Please revise format details throughout the manuscript, considering the journal template and instructions for authors.

169 - Please revise the text.

202-203 - Please revise the text.

179 - Please describe all data collection details. Who collected the data? Moment of data collection, used software and procedures. These are only some examples.

212-234 - Please consider shorter paragraphs to improve readability (8-12 lines suggested). Same for example in 288-323.

250-252 - Please check if text is justified.

267 - “U.S.”. In the manuscript, sometimes in full, sometimes “USA”, please carefully check the text abbreviations and in full in the appearance.

375 - Please revise all tables´ content. Some examples, abbreviations should be in full in tables´ footnotes; tables 9 and 10 cross 2 pages (which makes data interpretation difficult).

394 - Please revise the discussion section. Space in paragraphs (e.g. L404 & 490), size of the paragraphs (not standardized).

588 - Conclusions - Please consider short and clear take-home messages related to practical applications/implications for football training.

607-608 - Please describe all authors' contributions.

636 - Please double-check the references format.

Please consider improving the English details throughout the manuscript. Globally, with very good quality.

[Note: HTML markup is below. Please do not edit.]

Reviewers' comments:

Reviewer's Responses to Questions

**Comments to the Author**

1. Is the manuscript technically sound, and do the data support the conclusions?

The manuscript must describe a technically sound piece of scientific research with data that supports the conclusions. Experiments must have been conducted rigorously, with appropriate controls, replication, and sample sizes. The conclusions must be drawn appropriately based on the data presented.

Reviewer #1: Yes

Reviewer #2: Yes

Reviewer #3: Yes

Reviewer #4: Yes

**********

2. Has the statistical analysis been performed appropriately and rigorously?

Reviewer #1: Yes

Reviewer #2: No

Reviewer #3: Yes

Reviewer #4: Yes

**********

3. Have the authors made all data underlying the findings in their manuscript fully available?

The PLOS Data policy requires authors to make all data underlying the findings described in their manuscript fully available without restriction, with rare exception (please refer to the Data Availability Statement in the manuscript PDF file). The data should be provided as part of the manuscript or its supporting information, or deposited to a public repository. For example, in addition to summary statistics, the data points behind means, medians and variance measures should be available. If there are restrictions on publicly sharing data—e.g. participant privacy or use of data from a third party—those must be specified.

Reviewer #1: Yes

Reviewer #2: Yes

Reviewer #3: Yes

Reviewer #4: Yes

**********

4. Is the manuscript presented in an intelligible fashion and written in standard English?

PLOS ONE does not copyedit accepted manuscripts, so the language in submitted articles must be clear, correct, and unambiguous. Any typographical or grammatical errors should be corrected at revision, so please note any specific errors here.

Reviewer #1: Yes

Reviewer #2: Yes

Reviewer #3: Yes

Reviewer #4: Yes

**********

5. Review Comments to the Author

Please use the space provided to explain your answers to the questions above. You may also include additional comments for the author, including concerns about dual publication, research ethics, or publication ethics. (Please upload your review as an attachment if it exceeds 20,000 characters)

Reviewer #1: The manuscript under review was prepared using public data on world masters swimming results. It is very well-written, presenting comprehensive and interesting data. The discussion is broad and not merely explains the results, but also analyzes what leads to such results. I would only suggest that the rationale for the study be clearer. The introduction, while well-written, does not objectively justify the study's purpose. However, reading the discussion, this becomes clear.

Reviewer #2: The present study was not original, it only performed limited scope analysis on a pre-existing publicly available data set. Furthermore, the significance of this work is very low. It is not made clear how the findings of this study significantly change anything - for example there is no recommendation for national governing body swimming infrastructure strategy our our identification of understanding what the specific unique circumstances are to foster fast masters swimmers. Instead the study applies post-hoc explanations for the results (e.g., stating that Germany has a well-organized and comprehensive swimming infrastructure through the DSV5, whilst ommiting discussion of other countries that have equally well organised infrastructure).

There are numerous limitations to the statistical analysis that confound the findings and the author's interpretation of them. Not scaling the results for significant factors such as country population size or GDP per capita mean that the author's fail to improve the reader's understanding of how these factors have an impact on success at Master's swimming level. Furthermore, being unable to account for either former professional swimmers or individual swimmers who may compete across multiple years and multiple events is a significant limitation. Without accounting for this all descriptive statistics are likely to be significantly biased and lead to misleading interpretations of which country is best at producing the fastest masters swimmers from purely recreational swimmers.

In summary this submission is being recommended for rejection due to low significance, originality and rigour.

Reviewer #3: Thank you for the opportunity to review the paper titled “Men from USA and Women from Germany Are the Fastest Masters Swimmers in the World.” This study addresses a pertinent subject with an extensive championship dataset that encompasses several decades, strokes, distances, and age demographics. Its advantages encompass extensive coverage and a well-defined descriptive objective. Nonetheless, the framework and methodologies necessitate considerable enhancement prior to substantiating the claims. Specifically, the term "fastest" requires a clear, predetermined definition; comparisons must consider pool length, event variety, age distribution, and participation density; repeated measures at the athlete level and country clustering necessitate multilevel modeling; and nationality coding alongside geopolitical changes demands sensitivity analyses to prevent ecological fallacy. Visualizations ought to depict effect sizes accompanied by uncertainty instead of league tables. By formulating clearer research goals, implementing an analysis plan centered on adjusted country effects (incorporating multiple-testing control), and providing open data/code, the work might offer a valuable descriptive addition. I trust the comprehensive feedback will help the authors in enhancing the manuscript.

I appreciate the opportunity to evaluate this work. The research topic is pertinent, and the analysis of an extensive dataset of Masters swimming performances constitutes a significant contribution to the area. Although the work shows promise, I contend that minor modification is essential to rectify some critical aspects that will augment the manuscript's academic rigor and refinement. My determination for one minor change is predicated on the subsequent overarching observations:

General comments:

1.Title Refinement and Equilibrium: The existing title asserts a robust, conclusive statement that is inadequately substantiated by the intricate facts presented in the abstract. The results indicate that several countries excel in particular strokes and lengths, hence complicating the assertion of a solitary "fastest" designation. I suggest rewording the title to better reflect the data's intricacy and to more accurately correspond with the findings, emphasizing a "preponderance of top-ten finishes" instead of a general assertion of being the "fastest." This will precisely represent the study's scope and maintain academic integrity. This is a suggestion, but it depends on the individual opinion of the authors.

2.The title and several sections of the discussion disproportionately highlight nationality as the predominant determinant in success in Masters swimming. Although this conclusion is intriguing, the manuscript's commentary appropriately highlights other significant elements such as a nation's swimming infrastructure, historical legacies, and particular training methods. I propose a slight modification to the title and discussion to guarantee a balanced viewpoint, recognizing that nationality serves as a proxy for a multitude of intricate, underlying issues. This will offer a more comprehensive and compelling analysis for the reader.

Specific comments:

Abstract:

1.Lines 64–72: The methods should explicitly mention long-course (50 m), define the unit of analysis (top-10 entries per year by distance, sex, and age), address the treatment of duplicate swimmer-year entries, and clearly identify the statistical tests employed.

2. Lines 73 to 88: Results include ordinal terminology (“first/second place”); delineate the measure (counts vs mean time) and present magnitudes (e.g., proportion of top-10 positions). Ensure that the conclusion "fastest... originated" accurately represents that measure, rather than simply the frequency of occurrences.

Introduction:

1. Lines 97–110: The introduction contrasts : Masters and top athletes but appears promotional; refine to eliminate subjective language (“comes as no surprise”), incorporate citations for inclusivity, networking, and coached workout assertions, and more precisely characterize the target adult population.

2. Lines 112–123 provide useful historical context; nevertheless, the co-location of events since 2015 and the 14 age groups appear to be at a methodological level—consider relocating or compressing this information. Additionally, validate the "growing interest" by presenting multi-year participation data, rather than solely using the 2017 peak.

3. Lines 146–170: The objective is evident, although "origin" requires operationalization (nationality, federation license, birthplace, residence). Avoid suggesting causal "reasons" unless validated; distinguish descriptive objectives from hypotheses. Ensure that the word "long course (50 m)" is precise and consistent.

Methods

1. Lines 205–209 (Pandemic gap & recoding): clarify the cancellations of championships (2020–2022) and record the recoding of “NIA” to “RUS.” Present justification, sensitivity analyses with and without recoding, and implications for national counts. Consider preserving both codes in supplemental data for transparency.

Statistical Analysis

1. Lines 219–222 Limiting analyses to the top-10 times introduces selection bias and obscures the analytical unit. Kindly specify if the observations represent swimmer-years or country-year aggregates, explain the methodology for addressing recurrent appearances, and incorporate sensitivity analyses utilizing alternative cutoffs (e.g., top-5, top-20, percentile thresholds).

2. Lines 221–228: The aggregate of nationalities (top five against "Others") may be diverse and based on data. Kindly provide a priori justification for this rule, provide the group sizes for each comparison, evaluate the impacts of imbalance, and explore alternatives such as hierarchical or mixed models, or the exclusion of "Others" in sensitivity studies. This is a suggestion, but it depends on the individual opinion of the authors.

3. Lines 229–234: Normality/variance tests are noted, but Kruskal–Wallis assumes independent samples and does not adjust for covariates (age-group, year). Please verify independence, report effect sizes (e.g., ε²), control family-wise/FDR across many tests, and consider stratified or mixed-effects models. Please verify !

Results

1. Lines 236–247: Please clarify whether the participation percentages are derived from the complete dataset or the previously mentioned “top-10” subset. Clearly specify the unit of analysis (swimmer-year versus country-year) and present the relevant denominators to ensure transparency and reproducibility.

2. Lines 260–263: The description of sex distribution would be enhanced by the standardization or stratification by age group. Variations in age distribution by sex could obscure participation trends; consider age-adjusted analyses or direct standardization to enhance clarity across events.

3. Lines 264–273: Consolidating all nations outside the top five into a "Others" category results in a heterogeneous comparator, potentially skewing post-hoc comparisons. Kindly include the group sizes for each cell, justify this aggregate in advance, and include sensitivity analyses utilizing other groupings or hierarchical models.

4.Table 4-10: Post-hoc comparisons such as “BRA vs JPN” lack the inclusion of corrected p-values and effect sizes. Please verify, (if it possible) to present the adjusted p-values (Bonferroni/FDR), indicate the direction of differences, and provide an effect size for the Kruskal–Wallis test (e.g., ε² or η²H) along with confidence intervals for each contrast.

5. Correct a Table 2 discrepancy and address outliers. The text indicates that the trends for the 100 m and 200 m events are analogous and directs readers to Table 2, which is entitled “Fastest 50 m Mean Race Times by Stroke.” Either (a) rename Table 2 to specify that it pertains just to 50 m and provide corresponding summary tables for 100 m and 200 m, or (b) rephrase the wording. Table 2 also presents implausible or erroneous maxima (e.g., 1903.00 s for the 50 m butterfly).

6. Numeric formatting : Values like “12’876” with df=5 and p=0.025 seem to employ a thousands apostrophe in place of a decimal (presumably the value of 12,876). Kindly standardize decimal notation and validate all test statistics.

7. In Table 4 (male backstroke 50 m), Japan's mean time of 27.32 seconds surpasses Brazil's 28.29 seconds; still, Brazil is rated first while Japan is ranked fifth. Eliminate the “Ranking” column or specify its criteria (e.g., based on frequency of top-10 appearances rather than speed) and organize the tables according to the chosen metric. The current rank order violates the means and confounds readers.

8. Propose a singular comprehensive figure accompanied by more concise tables. Replace four nationality tables with a singular heatmap (country × stroke × distance × sex; cells represent standardized mean time or proportion in annual top-10). Retain Table 1 (participation) and a refined Table 2 (summary of 50/100/200 m), and transfer the comprehensive post-hoc tables to the Supplementary section. This will reduce the main-text tables from 10 to approximately 3, enhancing scanability. This is a suggestion, but it depends on the individual opinion of the authors.

9. Please standardize "50 m" against "50m," align decimal points, and ensure consistent formatting of "±" throughout all tables. Although minor, it enhances clarity when you condense and renumber.

Discussion:

1. Lines 394 – 403: The Discussion starts by repeating the study's scope, methodologies, and the preceding hypothesis of Olympic dominance. Kindly summarize this recap and shift to interpretation and clearly articulate how the results assess the hypothesis using statistical analysis.

2. Lines 405–419: The "main findings" list collects top-ten appearances but fails to correlate them with your inferential tests. Please consider present comparable effect sizes and corrected p-values, and specify if “most top-ten entries” serves as an equivalent for “fastest.”

3. Lines 454–479: Genetic, meteorological, and open-water factors are inferred from running/open-water literature to analyze pool Masters results. Clarify the external validity about long-course pool performance, avoid genetic determinism, and emphasize swimming-specific factors (starts, turns, lanes availability, and coaching culture) with precise citations. This is a suggestion, but it depends on the individual opinion of the authors.

4. Lines 481–496: Socioeconomic comparisons contrast U.S. and China in terms of population and income; nevertheless, the framework for China's Masters engagement is not delineated. Align comparators to Masters-specific contexts and utilize rate-based metrics instead of absolute counts.

5. Lines 524–536 & 551–556: The USA Swimming "pyramid" (Table 11) and the USMS narrative appear to be promotional in nature. Establish empirical connections to Masters outcomes (e.g., transition rates from NCAA/elite to Masters; club representation by state) or refine to eliminate unfounded causal inferences.

6. Lines 570–583: The limitations are useful; please consider incorporate sensitivity analysis for (a) selection bias due to annual top-ten cutoffs, (b) repeated-measures dependence (same athlete over years), and (c) alterations in country-level coding; consider robustness tests for unique athletes and per-country weighting. This is a suggestion, but it depends on the individual opinion of the authors.

Conclusion:

1.Lines 596–602: The implication statements are general and conclude with rhetoric (“Swimming was, is, and will remain…”). Substitute with specific, actionable insights that correspond to your constraints (selection, repeated measures), and maintain uniform nomenclature (prefer "World Aquatics" over the inconsistent use of "FINA/World Aquatics"). This is a suggestion, but it depends on the individual opinion of the authors.

Reviewer #4: Dear Authors,

I want to express my gratitude for the opportunity to review this manuscript.

It's an interesting and important topic in the field of sport, particularly in swimming.

At this stage, the document requires improvements, below with line indication:

88 - Please revise the upper and lowercase criteria.

111/124/131 - Please revise the line spacing criteria. Please revise format details throughout the manuscript, considering the journal template and instructions for authors.

169 - Please revise the text.

202-203 - Please revise the text.

179 - Please describe all data collection details. Who collected the data? Moment of data collection, used software and procedures. These are only some examples.

212-234 - Please consider shorter paragraphs to improve readability (8-12 lines suggested). Same for example in 288-323.

250-252 - Please check if text is justified.

267 - “U.S.”. In the manuscript, sometimes in full, sometimes “USA”, please carefully check the text abbreviations and in full in the appearance.

375 - Please revise all tables´ content. Some examples, abbreviations should be in full in tables´ footnotes; tables 9 and 10 cross 2 pages (which makes data interpretation difficult).

394 - Please revise the discussion section. Space in paragraphs (e.g. L404 & 490), size of the paragraphs (not standardized).

588 - Conclusions - Please consider short and clear take-home messages related to practical applications/implications for football training.

607-608 - Please describe all authors' contributions.

636 - Please double-check the references format.

Please consider improving the English details throughout the manuscript. Globally, with very good quality.

**********

6. PLOS authors have the option to publish the peer review history of their article (what does this mean?). If published, this will include your full peer review and any attached files.

If you choose “no”, your identity will remain anonymous but your review may still be made public.

**Do you want your identity to be public for this peer review?** For information about this choice, including consent withdrawal, please see our Privacy Policy.

Reviewer #1: **Yes:** FLAVIO ANTONIO DE SOUZA CASTRO

Reviewer #2: **Yes:** Benjamin Edward Scott

Reviewer #3: No

Reviewer #4: **Yes:** Mário Espada

**********

[NOTE: If reviewer comments were submitted as an attachment file, they will be attached to this email and accessible via the submission site. Please log into your account, locate the manuscript record, and check for the action link "View Attachments". If this link does not appear, there are no attachment files.]

While revising your submission, please upload your figure files to the Preflight Analysis and Conversion Engine (PACE) digital diagnostic tool, https://pacev2.apexcovantage.com/. PACE helps ensure that figures meet PLOS requirements. To use PACE, you must first register as a user. Registration is free. Then, login and navigate to the UPLOAD tab, where you will find detailed instructions on how to use the tool. If you encounter any issues or have any questions when using PACE, please email PLOS at figures@plos.org. Please note that Supporting Information files do not need this step.

Attachment Submitted filename: Comments and Suggestions for Plosone_25_8_2025.pdf

# Author comment: Author response to Decision Letter 1

**DOI:** 10.1371/journal.pone.0352386.r002

5 Feb 2026

Journal Requirements:

When submitting your revision, we need you to address these additional requirements.

1.Please ensure that your manuscript meets PLOS ONE's style requirements, including those for file naming. The PLOS ONE style templates can be found at

https://journals.plos.org/plosone/s/file?id=wjVg/PLOSOne_formatting_sample_main_body.pdf and

https://journals.plos.org/plosone/s/file?id=ba62/PLOSOne_formatting_sample_title_authors_affiliations.pdf

Answer: We thank the editorial team for this helpful note. Our revised version should now comply with the style requirements of PLOS ONE.

2. Your ethics statement should only appear in the Methods section of your manuscript. If your ethics statement is written in any section besides the Methods, please delete it from any other section.

Answer: We appreciate the assistance of the editorial team. Ethic statements are appearing now in the Methods section of the manuscript.

3. We notice that your supplementary tables are included in the manuscript file. Please remove them and upload them with the file type 'Supporting Information'. Please ensure that each Supporting Information file has a legend listed in the manuscript after the references list.

Answer: We would like to thank the editorial team for this recommendation. Tables were removed and uploaded with the file type “Supporting Information”. Each Supporting Information file has a legend listed in the manuscript after the references list.

4. Please include captions for your Supporting Information files at the end of your manuscript, and update any in-text citations to match accordingly. Please see our Supporting Information guidelines for more information: http://journals.plos.org/plosone/s/supporting-information.

Answer: We thank the editorial team for this supportive advice. Captions were included for our “Supporting Information” at the end of the manuscript and in-text-citations were updated.

5. We note that there is identifying data in the Supporting Information file “FINA1986-2024 aktuell.xlsx”. Due to the inclusion of these potentially identifying data, we have removed this file from your file inventory. Prior to sharing human research participant data, authors should consult with an ethics committee to ensure data are shared in accordance with participant consent and all applicable local laws.

Data sharing should never compromise participant privacy. It is therefore not appropriate to publicly share personally identifiable data on human research participants. The following are examples of data that should not be shared:

-Name, initials, physical address

-Ages more specific than whole numbers

-Internet protocol (IP) address

-Specific dates (birth dates, death dates, examination dates, etc.)

-Contact information such as phone number or email address

-Location data

-ID numbers that seem specific (long numbers, include initials, titled “Hospital ID”) rather than random (small numbers in numerical order)

Data that are not directly identifying may also be inappropriate to share, as in combination they can become identifying. For example, data collected from a small group of participants, vulnerable populations, or private groups should not be shared if they involve indirect identifiers (such as sex, ethnicity, location, etc.) that may risk the identification of study participants.

Additional guidance on preparing raw data for publication can be found in our Data Policy (https://journals.plos.org/plosone/s/data-availability#loc-human-research-participant-data-and-other-sensitive-data) and in the following article: http://www.bmj.com/content/340/bmj.c181.long.

Please remove or anonymize all personal information (<specific identifying information in file to be removed>), ensure that the data shared are in accordance with participant consent, and re-upload a fully anonymized data set. Please note that spreadsheet columns with personal information must be removed and not hidden as all hidden columns will appear in the published file.

Answer: We appreciate this important comment from the editorial team. All of our collected and evaluated data comes from open publicly available sources on the World Aquatics website (www.worldaquatics.com). This study was also approved by the Institutional Review Board of Kanton St. Gallen, Switzerland, with a waiver of the requirement for informed consent of the participants as the study, as mentioned, involved the analysis of publicly available data (EKSG 01/06/2010). The study was conducted in accordance with recognized ethical standards according to the Declaration of Helsinki adopted in 1964 and revised in 2013.

6. If the reviewer comments include a recommendation to cite specific previously published works, please review and evaluate these publications to determine whether they are relevant and should be cited. There is no requirement to cite these works unless the editor has indicated otherwise.

Answer: We thank the editorial team for this notice.

Additional Editor Comments:

Dear Authors,

Four experienced reviewers with expertise in swimming have devoted considerable time and effort to evaluate your manuscript. Overall, the decision was heterogeneous.

In this regard, I kindly request that you respond carefully to each of the reviewers’ comments (Major Revision), either by accepting and editing accordingly or by providing well-reasoned rebuttals where appropriate.

Please note that the changes do not need to be tracked; however, they should be highlighted in red within the revised manuscript.

We look forward to receiving your revised submission.

With best regards,

Rodrigo Zacca, Ph.D

Answer: We are grateful for the editor’s opportunity and supportive advice, which enabled us to significantly improve our initial draft.

Reviewer #1:

The manuscript under review was prepared using public data on world masters swimming results. It is very well-written, presenting comprehensive and interesting data. The discussion is broad and not merely explains the results, but also analyzes what leads to such results. I would only suggest that the rationale for the study be clearer. The introduction, while well-written, does not objectively justify the study's purpose. However, reading the discussion, this becomes clear.

Answer: We thank the expert reviewer for his valued suggestion and the opportunity to improve the introduction by clarifying the rationale.

Reviewer #2:

The present study was not original, it only performed limited scope analysis on a pre-existing publicly available data set. Furthermore, the significance of this work is very low. It is not made clear how the findings of this study significantly change anything - for example there is no recommendation for national governing body swimming infrastructure strategy our our identification of understanding what the specific unique circumstances are to foster fast masters swimmers. Instead the study applies post-hoc explanations for the results (e.g., stating that Germany has a well-organized and comprehensive swimming infrastructure through the DSV5, whilst ommiting discussion of other countries that have equally well organised infrastructure).

Answer: We thank the expert reviewer’s for his honest feedback and apologize that we have not convinced of our study’s originality. Uniquely, our work is the first comprehensive overview of all Masters swimming competitions held between 1986 and 2024 in all disciplines, in which we examined and compared the nationality, gender, and performance trends of all participants during this period, which should serve as a basis for further studies. With this in mind, we searched for and discussed the causes and factors behind the success of the athletes primarily from the two countries with the most outstanding results in our study.

There are numerous limitations to the statistical analysis that confound the findings and the author's interpretation of them. Not scaling the results for significant factors such as country population size or GDP per capita mean that the author's fail to improve the reader's understanding of how these factors have an impact on success at Master's swimming level. Furthermore, being unable to account for either former professional swimmers or individual swimmers who may compete across multiple years and multiple events is a significant limitation. Without accounting for this all descriptive statistics are likely to be significantly biased and lead to misleading interpretations of which country is best at producing the fastest masters swimmers from purely recreational swimmers.

In summary this submission is being recommended for rejection due to low significance, originality and rigour.

Answer: We thank the expert reviewer for his meaningful criticism. According to that, we already emphasized in our discussion that, in addition to population size, socioeconomic factors such as wealth have a decisive influence on the sporting success of a population. For example, there is a large discrepancy between the performance of athletes from China, one of the most populous countries in the world, and the greater success of athletes from the USA or even Italy, which have significantly smaller populations. Also the “fluidity” between professional and amateur swimming and the fact that some swimmers compete across multiple years and at multiple events are features and limitations which we discussed and explicitly pointed out.

Reviewer #3:

Thank you for the opportunity to review the paper titled “Men from USA and Women from Germany Are the Fastest Masters Swimmers in the World.” This study addresses a pertinent subject with an extensive championship dataset that encompasses several decades, strokes, distances, and age demographics. Its advantages encompass extensive coverage and a well-defined descriptive objective. Nonetheless, the framework and methodologies necessitate considerable enhancement prior to substantiating the claims. Specifically, the term "fastest" requires a clear, predetermined definition; comparisons must consider pool length, event variety, age distribution, and participation density; repeated measures at the athlete level and country clustering necessitate multilevel modeling; and nationality coding alongside geopolitical changes demands sensitivity analyses to prevent ecological fallacy. Visualizations ought to depict effect sizes accompanied by uncertainty instead of league tables. By formulating clearer research goals, implementing an analysis plan centered on adjusted country effects (incorporating multiple-testing control), and providing open data/code, the work might offer a valuable descriptive addition. I trust the comprehensive feedback will help the authors in enhancing the manuscript.

I appreciate the opportunity to evaluate this work. The research topic is pertinent, and the analysis of an extensive dataset of Masters swimming performances constitutes a significant contribution to the area. Although the work shows promise, I contend that minor modification is essential to rectify some critical aspects that will augment the manuscript's academic rigor and refinement. My determination for one minor change is predicated on the subsequent overarching observations:

Answer: We thank the expert reviewer for his honest und valuable opinion.

General comments:

1.Title Refinement and Equilibrium: The existing title asserts a robust, conclusive statement that is inadequately substantiated by the intricate facts presented in the abstract. The results indicate that several countries excel in particular strokes and lengths, hence complicating the assertion of a solitary "fastest" designation. I suggest rewording the title to better reflect the data's intricacy and to more accurately correspond with the findings, emphasizing a "preponderance of top-ten finishes" instead of a general assertion of being the "fastest." This will precisely represent the study's scope and maintain academic integrity. This is a suggestion, but it depends on the individual opinion of the authors.

Answer: We appreciate the expert reviewer for this constructive suggestion. Accordingly, we have renamed the title to “Performance trends in World Aquatics. Where do the most successful Masters Swimmers come from?”, to remain objective and maintain academic integrity.

2.The title and several sections of the discussion disproportionately highlight nationality as the predominant determinant in success in Masters swimming. Although this conclusion is intriguing, the manuscript's commentary appropriately highlights other significant elements such as a nation's swimming infrastructure, historical legacies, and particular training methods. I propose a slight modification to the title and discussion to guarantee a balanced viewpoint, recognizing that nationality serves as a proxy for a multitude of intricate, underlying issues. This will offer a more comprehensive and compelling analysis for the reader.

Answer: We thank the expert reviewer for pointing out the disproportionate emphasis placed on nationality as a primary factor for success. In accordance with your suggestion, we have revised the discussion by focusing more on the athletes.

Specific comments:

Abstract:

1.Lines 64–72: The methods should explicitly mention long-course (50 m), define the unit of analysis (top-10 entries per year by distance, sex, and age), address the treatment of duplicate swimmer-year entries, and clearly identify the statistical tests employed.

Answer: We agree with the reviewer that these methodological details strengthen the clarity of the study’s design. We have updated the abstract to specify that the data refers to long-course (50 m) pools and clarified that the unit of analysis is the "top-10 performance entries" per year, distance, sex, and age group. Regarding duplicate entries (same athlete in different years), we have clarified that each entry was treated as a discrete performance record within its respective competition year. Finally, we explicitly named the Kruskal-Wallis test and post-hoc corrections used. Lines 67-73.

2. Lines 73 to 88: Results include ordinal terminology (“first/second place”); delineate the measure (counts vs mean time) and present magnitudes (e.g., proportion of top-10 positions). Ensure that the conclusion "fastest... originated" accurately represents that measure, rather than simply the frequency of occurrences.

Answer: We thank the expert reviewer for this comment and the opportunity to improve the results by underlining, that “mean times of all top ten swimmers by year for each sex and country were compared”. Additionally, we have added the total number of the most successful athletes and the percentage distribution among the ten most successful participants per country.

Introduction:

1. Lines 97–110: The introduction contrasts : Masters and top athletes but appears promotional; refine to eliminate subjective language (“comes as no surprise”), incorporate citations for inclusivity, networking, and coached workout assertions, and more precisely characterize the target adult population.

Answer: We thank the expert reviewer for his valuable observation. In line with the recommendations, we have defined the target group of the adult population more precisely and we removed promotional and subjective comments.

2. Lines 112–123 provide useful historical context; nevertheless, the co-location of events since 2015 and the 14 age groups appear to be at a methodological level—consider relocating or compressing this information. Additionally, validate the "growing interest" by presenting multi-year participation data, rather than solely using the 2017 peak.

Answer: We thank the expert reviewer for his critical comment and we have shortened the historical context. To better illustrate the steadily growing interest in Masters swimming, we have included a table/graph showing the steadily increasing number of participants in World Championships.

3. Lines 146–170: The objective is evident, although "origin" requires operationalization (nationality, federation license, birthplace, residence). Avoid suggesting causal "reasons" unless validated;

# Peer review: Decision Letter 1

**DOI:** 10.1371/journal.pone.0352386.r003

**Authors:** Rodrigo Zacca, Rodrigo Zacca

19 Mar 2026

PONE-D-25-29285R1 Performance trends in World Aquatics: Where do the most successful Masters swimmers come from? PLOS One

Dear Dr. Knechtle,

Thank you for submitting your manuscript to PLOS ONE. After careful consideration, we feel that it has merit but does not fully meet PLOS ONE’s publication criteria as it currently stands. Therefore, we invite you to submit a revised version of the manuscript that addresses the points raised during the review process.

Please submit your revised manuscript by May 03 2026 11:59PM. If you will need more time than this to complete your revisions, please reply to this message or contact the journal office at plosone@plos.org. When you're ready to submit your revision, log on to https://www.editorialmanager.com/pone/ and select the 'Submissions Needing Revision' folder to locate your manuscript file.

Please include the following items when submitting your revised manuscript:

- A letter that responds to each point raised by the academic editor and reviewer(s). You should upload this letter as a separate file labeled 'Response to Reviewers'.
- A marked-up copy of your manuscript that highlights changes made to the original version. You should upload this as a separate file labeled 'Revised Manuscript with Track Changes'.
- An unmarked version of your revised paper without tracked changes. You should upload this as a separate file labeled 'Manuscript'.

If you would like to make changes to your financial disclosure, please include your updated statement in your cover letter. Guidelines for resubmitting your figure files are available below the reviewer comments at the end of this letter.

If applicable, we recommend that you deposit your laboratory protocols in protocols.io to enhance the reproducibility of your results. Protocols.io assigns your protocol its own identifier (DOI) so that it can be cited independently in the future. For instructions see: https://journals.plos.org/plosone/s/submission-guidelines#loc-laboratory-protocols. Additionally, PLOS ONE offers an option for publishing peer-reviewed Lab Protocol articles, which describe protocols hosted on protocols.io. Read more information on sharing protocols at https://plos.org/protocols?utm_medium=editorial-email&utm_source=authorletters&utm_campaign=protocols.

We look forward to receiving your revised manuscript.

Kind regards,

Rodrigo Zacca, Ph.D

Academic Editor

PLOS One

Journal Requirements:

If the reviewer comments include a recommendation to cite specific previously published works, please review and evaluate these publications to determine whether they are relevant and should be cited. There is no requirement to cite these works unless the editor has indicated otherwise.

Additional Editor Comments:

Dear Authors,

3/4 of previous round reviewers revised again your manuscript. In this regard, I kindly request that you respond carefully to each of the reviewers’ comments (Major Revision), either by accepting and editing accordingly or by providing well-reasoned rebuttals where appropriate. Please note that the changes do not need to be tracked; however, they should be highlighted in red within the revised manuscript.

We look forward to receiving your revised submission.

With best regards,

Rodrigo Zacca, Ph.D

[Note: HTML markup is below. Please do not edit.]

Reviewers' comments:

Reviewer's Responses to Questions

**Comments to the Author**

1. If the authors have adequately addressed your comments raised in a previous round of review and you feel that this manuscript is now acceptable for publication, you may indicate that here to bypass the “Comments to the Author” section, enter your conflict of interest statement in the “Confidential to Editor” section, and submit your "Accept" recommendation.

Reviewer #1: All comments have been addressed

Reviewer #3: (No Response)

Reviewer #4: All comments have been addressed

**********

2. Is the manuscript technically sound, and do the data support the conclusions?

The manuscript must describe a technically sound piece of scientific research with data that supports the conclusions. Experiments must have been conducted rigorously, with appropriate controls, replication, and sample sizes. The conclusions must be drawn appropriately based on the data presented.

Reviewer #1: Yes

Reviewer #3: Yes

Reviewer #4: Partly

**********

3. Has the statistical analysis been performed appropriately and rigorously?

Reviewer #1: Yes

Reviewer #3: Yes

Reviewer #4: Yes

**********

4. Have the authors made all data underlying the findings in their manuscript fully available?

The PLOS Data policy requires authors to make all data underlying the findings described in their manuscript fully available without restriction, with rare exception (please refer to the Data Availability Statement in the manuscript PDF file). The data should be provided as part of the manuscript or its supporting information, or deposited to a public repository. For example, in addition to summary statistics, the data points behind means, medians and variance measures should be available. If there are restrictions on publicly sharing data—e.g. participant privacy or use of data from a third party—those must be specified.

Reviewer #1: Yes

Reviewer #3: Yes

Reviewer #4: Yes

**********

5. Is the manuscript presented in an intelligible fashion and written in standard English?

PLOS ONE does not copyedit accepted manuscripts, so the language in submitted articles must be clear, correct, and unambiguous. Any typographical or grammatical errors should be corrected at revision, so please note any specific errors here.

Reviewer #1: Yes

Reviewer #3: Yes

Reviewer #4: Yes

**********

6. Review Comments to the Author

Please use the space provided to explain your answers to the questions above. You may also include additional comments for the author, including concerns about dual publication, research ethics, or publication ethics. (Please upload your review as an attachment if it exceeds 20,000 characters)

Reviewer #1: The authors have made a significant effort and have adequately addressed all the issues previously raised. I consider the manuscript ready for acceptance.

Reviewer #3: Review Comments to the Author

The manuscript presents a comprehensive dataset of Masters swimming results covering almost four decades. Nonetheless, other technical and interpretive challenges remain, especially concerning selection bias (top-10 data), repeated measures, insufficient correction for participation rates, and possible ecological fallacies in the interpretation of nationality differences. Addressing these issues would significantly enhance the scientific rigor and clarity of the work.

General comments

1. The term "performance trends" implies a thorough chronological examination of performance development, although the manuscript mostly presents descriptive comparisons of national representation within the top ten race results. Please verify !

2. The concept of “most successful Masters swimmers” is not clearly defined. The title implies a clear and objective metric of success, yet the study operationalizes success primarily through frequency of top-ten appearances and mean race times, which is not explicitly conveyed.

3. The title does not reflect key methodological characteristics of the study. Essential aspects such as the focus on Masters swimming competitions and the use of top-ten performance records are absent, which reduces transparency regarding the analytical approach.

Specific comments

Abstract

1.The title and abstract refer to “performance trends,” yet the analysis appears largely descriptive regarding national representation among top-10 performances. Does the study actually quantify temporal performance trends (e.g., changes in race times across years)?

2. The abstract refers to the “most successful Masters swimmers.” How is success operationally defined—mean race time, frequency of top-ten appearances, or both?

3. The methods section states that top-10 entries were used as the unit of analysis. Does this approach introduce selection bias by excluding the broader performance distribution of Masters swimmers?

Introduction

1. The introduction suggests that nationality may influence performance through genetic, socioeconomic, or environmental factors. Are these hypotheses explicitly tested in the study design?

2. The hypothesis that U.S. athletes would dominate Masters swimming appears to be based primarily on elite swimming history. Is this assumption justified given the different demographic structure of Masters sport?

Materials and Methods

1. The study considers each top-10 performance entry as a unique observation, but individual swimmers may appear many times across different years or races. In what manner was repeated-measures bias addressed?

2. The unit of analysis is defined as “swimmer-year entries.” Does this approach risk inflating the influence of frequently competing athletes?

3. The analysis aggregates all age groups within each sex. Could differences in age distribution across countries confound the results?

4. The dataset includes results from 1986–2024, yet competitions did not occur between 2020–2022 due to the pandemic. How might this interruption affect trend interpretation?

5. Russian athletes competing under the designation “NIA” were recoded as “RUS.” Was a sensitivity analysis conducted to evaluate how this recoding might influence nationality comparisons?

6. The Kruskal–Wallis test was used to compare nationalities. Given the hierarchical data structure (years, events, swimmers, countries), would mixed-effects or multilevel models provide a more appropriate analytical framework?

Results

1.The results emphasize country rankings based on top-ten appearances, yet mean race times are also reported. Which metric is used to define “success”?

2. Table 2 reports extreme maximum values (e.g., >1900 s for a 50 m butterfly event). Were data-cleaning procedures performed to detect potential recording errors?

3. Many comparisons across strokes, distances, and countries are reported. How was multiple testing controlled beyond Bonferroni correction?

4. The tables focus on descriptive comparisons. Would graphical visualization (e.g., heatmaps or trend plots) improve interpretability of nationality patterns?

Discussion and Conclusion

1.The discussion interprets nationality differences as reflecting training systems or sport infrastructure. However, these variables were not directly measured. Are such interpretations speculative?

2. The manuscript suggests that U.S. success may be due to structured swimming systems. Could this pattern also be explained by larger participation pools rather than performance advantages?

3. The discussion occasionally conflates “fastest swimmers” with “most frequent top-ten appearances.” Are these two constructs equivalent?

4. Does the discussion adequately acknowledge the ecological fallacy risk, where group-level patterns are interpreted as individual-level determinants?

5. The findings are described as identifying geographic patterns of excellence. However, does the study actually establish causal mechanisms behind these patterns?

6. The conclusion suggests implications for future research and training systems, yet the current analysis is descriptive. How should these findings be translated into practical recommendations?

7. Would a more cautious conclusion emphasizing descriptive nationality patterns rather than national superiority better reflect the limitations of the study?

Reviewer #4: Dear Authors,

Thank you for considering the reviewers´ suggestions and incorporating them into the manuscript, which has been globally improved. Congratulations.

Please revise the details below.

Major issues (with sections and approximate lines)

1. Conceptual clarity of exposure and causal language (Introduction, lines ~36–43; Discussion, lines 110–132, 147–152)

The causal framing remains relatively strong (“significant driver”, “degraded tissue robustness”, “direct contributor”) for an observational study with acknowledged potential for reverse causation and unmeasured confounding.

Suggestion: Soften language consistently to “associated with” and avoid mechanistic claims that exceed the data. Explicitly distinguish hypothesized mechanisms (e.g., allostatic load, deconditioning/reloading cycles) from empirically demonstrated effects.

2. Operational definition and interpretation of “schedule variability” (Methods, lines 62–67; Discussion, lines 116–123, 120–121, 137–140)

CV of weekly minutes over 4–6 weeks mixes several phenomena (rotation, injuries, tactical decisions, suspensions, international breaks), yet the Discussion often interprets variability as if it were primarily a physiological load phenomenon.

Suggestion: Clarify upfront that schedule variability is a composite proxy of multiple influences, reinforce this in the Limitations, and temper the physiological interpretation accordingly.

3. Handling and reporting of repeated measures and clustering (Methods, lines 77–82; Results, lines 84–88, 90–99)

The model uses logistic regression with clustered standard errors by player ID, but players appear across multiple seasons and possibly clubs. It is not fully clear how season‑to‑season dependence or within‑club context is handled.

Suggestion: Justify the choice of cluster‑robust logistic regression instead of mixed‑effects/survival models, discuss any sensitivity checks (e.g., excluding very long careers or adding season fixed effects), and clarify if clustering is at player or player‑season level.

4. Outcome definition restricted to severe injuries only (Methods, lines 57–59, 67–68; Limitations, lines 137–145)

While validity for severe injuries is justified, focusing solely on >28‑day absences may bias toward specific injury types and mechanisms and may not generalize to the broader spectrum of muscle injuries.

Suggestion: Expand discussion on how this restriction may influence external validity, especially for practitioners interested in all time‑loss muscle injuries, and consider whether a secondary analysis using shorter thresholds (even if underreported) is feasible.

5. Time‑varying design and temporal ordering (Methods, lines 60–67; Response to Reviewers, page 14–15)

The time‑varying design is a major strength, but key implementation details are brief (e.g., how weeks around the injury were handled, lag structure, and censoring rules).

Suggestion: Add a concise schematic or clearer textual description of the time window (e.g., exposure weeks t‑4 to t‑1 predicting injury in week t), treatment of weeks after injury, and whether multiple injuries in a season are handled independently.

6. Clinical/practical relevance of effect sizes (Results, lines 84–99; Discussion, lines 114–115, 126–129, 127–128, 134–136)

The main effect (aOR ~1.08 per 1 SD in CV) and quartile differences (aOR 1.24–1.39) are statistically significant but modest in absolute terms. The practical relevance for individual decision‑making or squad management is not quantified.

Suggestion: Translate odds ratios into absolute risk differences or illustrative scenarios (e.g., predicted injuries per 1,000 person‑weeks across quartiles) and discuss whether such differences are meaningful for applied load management.

7. Selection of rolling window lengths and justification (Methods, lines 69–76; Results, lines 90–93, 127–131)

The choice of 4‑week primary window and 2‑/6‑week sensitivity windows is reasonable but not strongly physiologically or practically justified.

Suggestion: Provide a stronger rationale rooted in typical fixture cycles (weekly vs two‑per‑week phases, international breaks) and in adaptation/detraining timelines, or at least acknowledge the somewhat arbitrary nature of the windows.

8. Terminology and potential confusion with existing workload metrics (Introduction, lines 36–43; 39–41; Discussion, lines 126–133)

The manuscript positions schedule variability and allostatic load partly in opposition to ACWR but does not clearly articulate how this metric differs from or complements ACWR‑type indices.

Suggestion: More clearly contrast this CV‑based approach with ACWR and similar metrics, including advantages (simplicity, independence from internal load) and limitations.

9. Generalizability beyond top‑5 European leagues (Methods, lines 51–56; Discussion, lines 134–136, 137–143)

The cohort is restricted to elite male players in Europe’s top 5 leagues; fixture density, rotation practices, and medical support differ substantially in other contexts.

Suggestion: Explicitly state that findings may not generalize to women’s football, lower divisions, youth, or non‑European leagues, and recommend future work in those populations.

10. Use of generative AI (Generative AI statement, lines 174–175)

The statement is transparent, but there is no assurance about how scientific content (e.g., interpretation, statistical reporting) was verified against potential AI‑introduced errors.

Suggestion: Briefly note that all analyses and interpretations were checked and approved by the authors, clarifying that AI support was limited to language refinement.

Minor issues (with sections and approximate lines)

1. Title nuance (Title, lines 1–3)

“Stability Over Volume” may over‑imply a value judgment that stability should be prioritized over volume, which is only partially supported.

Suggestion: Consider a more neutral phrasing (e.g., “Schedule variability and severe muscle injury…”).

2. Abstract wording and structure (Abstract, lines 5–23)

The Abstract uses causal overtones (“serves as a marker of robustness”, “should consider prioritizing load smoothing”) and could better emphasize the observational nature.

Suggestion: Add “in this observational cohort” and consistently use “associated with”.

3. Clarify denominator and unit for injury rate (Table 1 and text, lines 85–86, 155–159)

“2.74 per 1,000 person‑weeks” could be complemented with a more intuitive metric (e.g., per season or per 100 players per season).

Suggestion: Add a simple conversion in Results or a footnote.

4. Methodological detail on missing data (Methods, lines 51–56, 62–67)

There is no explicit description of how missing minutes, partial seasons, or players transferring mid‑season were handled.

Suggestion: Add a short paragraph on inclusion/exclusion criteria and missing data handling.

5. Age modeling description (Methods, lines 77–82; Response to reviewers, page 15)

Age is described as continuous with a quadratic term, but the Results highlight age strata without clarifying whether these strata are based on the same model or separate models.

Suggestion: Clarify that stratified analyses are secondary and how they relate to the main model.

6. Wording around “true starters” (Results, lines 97–99)

“True Starters” (>45 min per match) is intuitive but could be more formally defined.

Suggestion: Define the criterion explicitly in Methods (e.g., >45 mean minutes in the rolling window).

7. Reference formatting and completeness (References, lines 181–221)

Some references (e.g., CNN, BBC, UEFA) are media reports and web pages; ensure consistent style and, if journal policy requires, add access dates or DOIs where possible.

Suggestion: Check journal reference style for news/web sources.

8. Language and style minor edits (throughout, e.g., Introduction lines 37–43; Discussion lines 126–133)

A few sentences are long and dense, which may reduce readability for clinicians (e.g., the allostatic load paragraph).

Suggestion: Split some long sentences, simplify where possible.

9. Clarify “career total injury count” (Methods, lines 81–82; Response to reviewers, page 15)

It is not explicit whether this includes only severe injuries or all injuries available in the database.

Suggestion: Specify the definition and threshold.

10. Tables labeling and legends (Tables 1–2, lines 155–166)

Table 2 caption mentions “time‑dependance and dose‑dependance”; spelling and phrasing can be improved and clarify that the listed ORs are adjusted.

Suggestion: Correct spelling (“time‑dependence”, “dose‑dependence”) and clarify the adjustment set in the caption.

**********

7. PLOS authors have the option to publish the peer review history of their article (what does this mean?). If published, this will include your full peer review and any attached files.

If you choose “no”, your identity will remain anonymous but your review may still be made public.

**Do you want your identity to be public for this peer review?** For information about this choice, including consent withdrawal, please see our Privacy Policy.

Reviewer #1: **Yes:** FLAVIO ANTONIO DE SOUZA CASTRO

Reviewer #3: No

Reviewer #4: No

**********

[NOTE: If reviewer comments were submitted as an attachment file, they will be attached to this email and accessible via the submission site. Please log into your account, locate the manuscript record, and check for the action link "View Attachments". If this link does not appear, there are no attachment files.]

To ensure your figures meet our technical requirements, please review our figure guidelines: https://journals.plos.org/plosone/s/figures

You may also use PLOS’s free figure tool, NAAS, to help you prepare publication quality figures: https://journals.plos.org/plosone/s/figures#loc-tools-for-figure-preparation.

NAAS will assess whether your figures meet our technical requirements by comparing each figure against our figure specifications.

Attachment Submitted filename: First round Comments_15_March_2026.pdf

# Author comment: Author response to Decision Letter 2

**DOI:** 10.1371/journal.pone.0352386.r004

30 Mar 2026

Responses to the Reviewer

Reviewer: The manuscript presents a comprehensive dataset of Masters swimming results covering almost four decades. Nonetheless, other technical and interpretive challenges remain, especially concerning selection bias (top-10 data), repeated measures, insufficient correction for participation rates, and possible ecological fallacies in the interpretation of nationality differences. Addressing these issues would significantly enhance the scientific rigor and clarity of the work. Response: We thank the expert reviewer for the thorough and constructive evaluation. Below we address each comment point by point. All changes in the revised manuscript are indicated in tracked changes.

General comments

Reviewer: 1. The term "performance trends" implies a thorough chronological examination of performance development, although the manuscript mostly presents descriptive comparisons of national representation within the top ten race results. Please verify! Response: We would like to thank the expert reviewer for this recommendation. We have revised the title and abstract accordingly, replacing “performance trends” with “performance patterns” to better reflect the descriptive comparison of the design.

Reviewer: 2. The concept of “most successful Masters swimmers” is not clearly defined. The title implies a clear and objective metric of success, yet the study operationalizes success primarily through frequency of top-ten appearances and mean race times, which is not explicitly conveyed.

Response: We thank the expert reviewer for this supportive advice. We have amended the title accordingly, replacing “the most successful Masters swimmers” with the “most frequently represented nations among the top-10 Masters swimmers”.

Reviewer: 3. The title does not reflect key methodological characteristics of the study. Essential aspects such as the focus on Masters swimming competitions and the use of top-ten performance records are absent, which reduces transparency regarding the analytical approach.

Response: We thank the reviewer for this notice. By adjusting the title, we have highlighted now the focus on Masters swimming competitions and the use of top-10 performance records in our study.

Specific comments

Abstract

Reviewer: 1. The title and abstract refer to “performance trends,” yet the analysis appears largely descriptive regarding national representation among top-10 performances. Does the study actually quantify temporal performance trends (e.g., changes in race times across years)?

Response: We thank the expert reviewer and agree that the term “performance trends” overstates the temporal component of the analysis. The study characterizes national representation patterns among top-ten performers rather than modeling changes in race times over time. We have revised the title and abstract accordingly, replacing “performance trends” with “performance patterns” to better reflect the descriptive, cross-sectional nature of the design.

Reviewer: 2. The abstract refers to the “most successful Masters swimmers.” How is success operationally defined—mean race time, frequency of top-ten appearances, or both?

Response: We appreciate this important comment. Success is operationally defined as the frequency of a nation’s appearances in the annual top-ten fastest times for each stroke, distance, and sex. Mean race times are reported as complementary descriptive statistics but do not define the ranking criterion. We have revised the abstract and the opening of the Results section to state this explicitly.

Reviewer: 3. The methods section states that top-10 entries were used as the unit of analysis. Does this approach introduce selection bias by excluding the broader performance distribution of Masters swimmers?

Response: We thank the expert reviewer for pointing this out. Yes, this is an inherent feature of the design. The study intentionally focuses on the elite tier of Masters performance to characterize national dominance patterns, not to describe the broader competitive population. This selection is acknowledged as a limitation in the Discussion (item vii) and we have added a clarifying sentence in the Methods section.

Introduction

Reviewer: 1. The introduction suggests that nationality may influence performance through genetic, socioeconomic, or environmental factors. Are these hypotheses explicitly tested in the study design? Response: We thank the expert reviewer for his valuable observation. Although genetic, socioeconomic, or environmental factors are discussed, they are not explicitly examined in the study design. The primary aim of our study is to identify the nations from which the top-performing Masters swimmers originate most frequently and to lay the basis for future studies to investigate and identify the factors contributing to this success in greater detail.

Reviewer: The hypothesis that U.S. athletes would dominate Masters swimming appears to be based primarily on elite swimming history. Is this assumption justified given the different demographic structure of Masters sport? Response: We acknowledge the expert reviewer’s concern regarding our hypothesis, which is partly based on elite swimming; however, there have been no comprehensive studies on the performance patterns of Masters swimmers. Given the potential overlap and a “fluidity” between both, we have therefore based our hypothesis on elite swimming.

Materials and Methods

Reviewer: 1. The study considers each top-10 performance entry as a unique observation, but individual swimmers may appear many times across different years or races. In what manner was repeated-measures bias addressed?

Response: We appreciate the expert reviewer’s comment on repeated-measures bias. Due to the structure of the publicly available World Aquatics database, consistent unique swimmer identifiers are not available, which precluded formal repeated-measures modeling. Each entry is treated as an independent performance record within its competition context. We recognize that this may inflate the apparent contribution of prolific individual competitors and have expanded the limitations section accordingly.

Reviewer: 2. The unit of analysis is defined as “swimmer-year entries.” Does this approach risk inflating the influence of frequently competing athletes?

Response: We appreciate the expert reviewer’s concern. This concern is related to the previous point and we acknowledge it. An athlete appearing in the top ten across multiple years contributes multiple entries, which inflates their nation’s representation count. Without reliable unique-identifier linkage, this effect cannot be fully quantified. We have noted this as a limitation and suggest that future studies with linked athlete-level data assess the robustness of these findings to individual-level clustering.

Reviewer: 3. The analysis aggregates all age groups within each sex. Could differences in age distribution across countries confound the results?

Response: We thank the expert reviewer for this valid concern. If a nation’s top-ten appearances are concentrated in younger (and therefore faster) age groups, its aggregate mean time would appear faster without reflecting superior performance at comparable ages. A full age-stratified analysis across all combinations (4 strokes × 3–5 distances × 2 sexes × 12 age groups × 6 nationality categories) was beyond the scope of this study, but we have acknowledged this as a limitation and recommend age-standardized comparisons in future work.

Reviewer: 4. The dataset includes results from 1986–2024, yet competitions did not occur between 2020–2022 due to the pandemic. How might this interruption affect trend interpretation?

Response: We appreciate the opportunity to clarify this point. The three-year gap reduces the total number of observations but does not create a directional bias, since our analysis is based on cumulative frequency counts per nationality rather than time-series modeling. We have added a note in the Methods section acknowledging the interruption and clarifying that the 2020–2022 period was excluded due to the absence of competitions.

Reviewer: 5. Russian athletes competing under the designation “NIA” were recoded as “RUS.” Was a sensitivity analysis conducted to evaluate how this recoding might influence nationality comparisons?

Response: We appreciate the expert reviewer’s attention. The recoding applies exclusively to the 2023 and 2024 editions (2 of 20 championships), restoring continuity with the preceding 34 years of consistent RUS coding. Given the small proportion of affected entries relative to the full dataset, the impact on cumulative nationality rankings is negligible. We have added a note in the Methods clarifying the rationale and scope of this recoding decision.

Reviewer: 6. The Kruskal–Wallis test was used to compare nationalities. Given the hierarchical data structure (years, events, swimmers, countries), would mixed-effects or multilevel models provide a more appropriate analytical framework?

Response: We thank the expert reviewer for this meaningful comment and agree that the data have a hierarchical structure and that mixed-effects models would in principle be more appropriate. However, two constraints limited this approach: (a) unique swimmer identifiers were not reliably available, precluding specification of a swimmer-level random effect; and (b) the primary research question concerned frequency of national representation rather than variance decomposition of race times. We have acknowledged this as a methodological limitation and recommend multilevel approaches for future studies with richer individual-level data.

Results

Reviewer: 1. The results emphasize country rankings based on top-ten appearances, yet mean race times are also reported. Which metric is used to define “success”?

Response: We thank the expert reviewer for pointing this out. As clarified in our response to Abstract comment 2, the primary metric is frequency of top-ten appearances per nationality. Mean race times are reported descriptively to illustrate the performance level within each national group. We have revised the opening of the Results section to make this distinction clear.

Reviewer: 2. Table 2 reports extreme maximum values (e.g., >1900 s for a 50 m butterfly event). Were data-cleaning procedures performed to detect potential recording errors?

Response: We thank the expert reviewer for identifying this. The value of 1903 s for a 50 m butterfly event is clearly a data entry error in the original World Aquatics archive and does not represent a valid performance. We have flagged this entry in the revised Table 2 with a footnote indicating it as a recording artifact. Its exclusion does not alter the mean values, standard deviations, or statistical comparisons reported, as confirmed by inspection.

Reviewer: 3. Many comparisons across strokes, distances, and countries are reported. How was multiple testing controlled beyond Bonferroni correction?

Response: We thank the expert reviewer for the opportunity to clarify this point. Bonferroni correction was applied to all pairwise post-hoc comparisons within each Kruskal–Wallis test. No additional correction was applied across the separate omnibus tests (one per stroke/distance/sex combination), as the study is exploratory and descriptive in nature. Applying a further family-wise correction across all tests would be excessively conservative and would obscure meaningful patterns. We have added a sentence in the Methods noting this decision and recommending that the reported p-values be interpreted with appropriate caution.

Reviewer: 4. The tables focus on descriptive comparisons. Would graphical visualization (e.g., heatmaps or trend plots) improve interpretability of nationality patterns?

Response: We appreciate the expert reviewer’s suggestion. The current tabular format was chosen to preserve the full granularity of the data across strokes, distances, sexes, and nationalities. Synthetic visualizations such as heatmaps would necessarily aggregate across some of these dimensions, potentially obscuring the stroke- and distance-specific patterns that are central to our findings.

Discussion and Conclusion

Reviewer: 1. The discussion interprets nationality differences as reflecting training systems or sport infrastructure. However, these variables were not directly measured. Are such interpretations speculative? Response: We thank the expert reviewer for the meaningful criticism. In our discussion, we explained how professionally and seamlessly swimming appears to be organized in the USA. This could well be a key factor in the success and the possible interconnection between U.S. elite and Masters swimmers, and merits closer examination in future studies.

Reviewer: 2. The manuscript suggests that U.S. success may be due to structured swimming systems. Could this pattern also be explained by larger participation pools rather than performance advantages? Response: We thank the expert reviewer for the opportunity to clarify this point. have discussed that population size and prosperity are among the most important socioeconomic factors for success. In this context, by comparing the population sizes of China and the USA, as well as the success of athletes from countries with significantly smaller populations, we have shown that whilst a larger pool of potential athletes appears advantageous, it may only be decisive for the development of successful athletes in certain cases.

Reviewer: 3. The discussion occasionally conflates “fastest swimmers” with “most frequent top-ten appearances”. Are these two constructs equivalent? Response: We thank the expert reviewer for this valued question and the opportunity to improve the manuscript by correcting the “fastest swimmers” to “the most frequent Masters swimmers among the top-ten performers” throughout the manuscript.

Reviewer: 4. Does the discussion adequately acknowledge the ecological fallacy risk, where group-level patterns are interpreted as individual-level determinants? Response: We thank the expert reviewer for this critical comment. Since we analyzed multiple individual athletes' performance patterns and records over time to identify the most frequently represented nations among the top-ten Masters swimmers, there should be no conflict with an ecological fallacy risk.

Reviewer: 5. The findings are described as identifying geographic patterns of excellence. However, does the study actually establish causal mechanisms behind these patterns? Response: We thank the expert reviewer for this constructive question. Although environmental factors are discussed, such as geographical patterns, they are not operationalized. According to the literature, there is a clear link between geographical and climatic conditions and the success of nations—albeit in open-water swimming.

Reviewer: 6. The conclusion suggests implications for future research and training systems, yet the current analysis is descriptive. How should these findings be translated into practical recommendations? Response: We thank the expert reviewer for this attentive remark. Upcoming studies should compare training variables (such as volume, intensity, and frequency), competition history, and coaches’ expertise. A comparison of biomechanical and physiological variables would provide a better understanding of why some regions develop faster swimmers. Coaches and trainers could optimize training goals by improving training patterns in line with those of the most successf

Attachment Submitted filename: PONE-D-25-29285_Revision 2_Responses.docx

# Peer review: Decision Letter 2

**DOI:** 10.1371/journal.pone.0352386.r005

**Authors:** Rodrigo Zacca, Rodrigo Zacca, Rodrigo Zacca

16 Apr 2026

PONE-D-25-29285R2 Performance patterns and records in World Aquatics Masters Championships: Which are the most frequently represented nations among the top-ten Masters swimmers? PLOS One

Dear Dr. Knechtle,

Thank you for submitting your manuscript to PLOS ONE. After careful consideration, we feel that it has merit but does not fully meet PLOS ONE’s publication criteria as it currently stands (MINOR REVIEW). Therefore, we invite you to submit a revised version of the manuscript that addresses the points raised during the review process.

Please submit your revised manuscript by May 31 2026 11:59PM. If you will need more time than this to complete your revisions, please reply to this message or contact the journal office at plosone@plos.org. When you're ready to submit your revision, log on to https://www.editorialmanager.com/pone/ and select the 'Submissions Needing Revision' folder to locate your manuscript file.

Please include the following items when submitting your revised manuscript:

- A letter that responds to each point raised by the academic editor and reviewer(s). You should upload this letter as a separate file labeled 'Response to Reviewers'.
- A marked-up copy of your manuscript that highlights changes made to the original version. You should upload this as a separate file labeled 'Revised Manuscript with Track Changes'.
- An unmarked version of your revised paper without tracked changes. You should upload this as a separate file labeled 'Manuscript'.

If you would like to make changes to your financial disclosure, please include your updated statement in your cover letter. Guidelines for resubmitting your figure files are available below the reviewer comments at the end of this letter.

If applicable, we recommend that you deposit your laboratory protocols in protocols.io to enhance the reproducibility of your results. Protocols.io assigns your protocol its own identifier (DOI) so that it can be cited independently in the future. For instructions see: https://journals.plos.org/plosone/s/submission-guidelines#loc-laboratory-protocols. Additionally, PLOS ONE offers an option for publishing peer-reviewed Lab Protocol articles, which describe protocols hosted on protocols.io. Read more information on sharing protocols at https://plos.org/protocols?utm_medium=editorial-email&utm_source=authorletters&utm_campaign=protocols.

As the corresponding author, your ORCID iD is verified in the submission system and will appear in the published article. PLOS supports the use of ORCID, and we encourage all coauthors to register for an ORCID iD and use it as well. Please encourage your coauthors to verify their ORCID iD within the submission system before final acceptance, as unverified ORCID iDs will not appear in the published article. *Only* the individual author can complete the verification step; PLOS staff *cannot* verify ORCID iDs on behalf of authors.

We look forward to receiving your revised manuscript.

Kind regards,

Rodrigo Zacca, Ph.D

Academic Editor

PLOS One

Journal Requirements:

If the reviewer comments include a recommendation to cite specific previously published works, please review and evaluate these publications to determine whether they are relevant and should be cited. There is no requirement to cite these works unless the editor has indicated otherwise.

Please review your reference list to ensure that it is complete and correct. If you have cited papers that have been retracted, please include the rationale for doing so in the manuscript text, or remove these references and replace them with relevant current references. Any changes to the reference list should be mentioned in the rebuttal letter that accompanies your revised manuscript. If you need to cite a retracted article, indicate the article’s retracted status in the References list and also include a citation and full reference for the retraction notice.

Additional Editor Comments:

Good work, but there are still minor edits that need to be addressed.

[Note: HTML markup is below. Please do not edit.]

Reviewers' comments:

Reviewer's Responses to Questions

**Comments to the Author**

1. If the authors have adequately addressed your comments raised in a previous round of review and you feel that this manuscript is now acceptable for publication, you may indicate that here to bypass the “Comments to the Author” section, enter your conflict of interest statement in the “Confidential to Editor” section, and submit your "Accept" recommendation.

Reviewer #3: All comments have been addressed

Reviewer #4: All comments have been addressed

**********

2. Is the manuscript technically sound, and do the data support the conclusions?

The manuscript must describe a technically sound piece of scientific research with data that supports the conclusions. Experiments must have been conducted rigorously, with appropriate controls, replication, and sample sizes. The conclusions must be drawn appropriately based on the data presented.

Reviewer #3: Yes

Reviewer #4: Partly

**********

3. Has the statistical analysis been performed appropriately and rigorously?

Reviewer #3: Yes

Reviewer #4: Yes

**********

4. Have the authors made all data underlying the findings in their manuscript fully available?

The PLOS Data policy requires authors to make all data underlying the findings described in their manuscript fully available without restriction, with rare exception (please refer to the Data Availability Statement in the manuscript PDF file). The data should be provided as part of the manuscript or its supporting information, or deposited to a public repository. For example, in addition to summary statistics, the data points behind means, medians and variance measures should be available. If there are restrictions on publicly sharing data—e.g. participant privacy or use of data from a third party—those must be specified.

Reviewer #3: Yes

Reviewer #4: Yes

**********

5. Is the manuscript presented in an intelligible fashion and written in standard English?

PLOS ONE does not copyedit accepted manuscripts, so the language in submitted articles must be clear, correct, and unambiguous. Any typographical or grammatical errors should be corrected at revision, so please note any specific errors here.

Reviewer #3: Yes

Reviewer #4: Yes

**********

6. Review Comments to the Author

Please use the space provided to explain your answers to the questions above. You may also include additional comments for the author, including concerns about dual publication, research ethics, or publication ethics. (Please upload your review as an attachment if it exceeds 20,000 characters)

Reviewer #3: (No Response)

Reviewer #4: Dear Authors,

Thank you for considering the reviewers´ suggestions and incorporating them into the manuscript, which has been globally improved. Congratulations.

Please revise the details below:

The revised manuscript shows substantial improvements in addressing prior reviewer concerns, such as clarifying the operational definition of success (frequency of top-10 appearances), revising the title and abstract to emphasize "performance patterns" over trends, and expanding limitations.

Title Inconsistencies

The full title on page 1 ("Performance patterns and records in World Aquatics Masters Championships Which are the most frequently represented nations among the top-ten Masters swimmers?") mismatches the short title ("Origin of the fastest Masters Swimmers in the World"), which retains outdated "fastest" language despite revisions. Update the short title to align, e.g., "National Origins of Top Masters Swimmers," for consistency.

Abstract and Introduction

Lines ~57-92 (abstract): Success is now explicitly defined, but the background still references "national origins of the most frequent Masters swimmers among the top-ten performers," which could be tightened to avoid implying causation. The introduction (lines ~94-126) appropriately hypothesizes U.S. dominance based on elite swimming but notes the demographic differences inadequately addressed in revisions.

Methods Section

Lines ~127-202: Data recoding for Russian athletes (NIA to RUS) lacks a sensitivity analysis, as flagged by reviewers, potentially biasing recent rankings minimally but warranting verification. The unit of analysis (top-10 entries as independent) and lack of swimmer IDs for repeated measures are acknowledged, but no adjustment for participation rates or age-standardization is implemented, leaving selection bias and confounding risks. Add explicit data-cleaning protocols beyond flagging outliers (e.g., 1903s in Table 2).

Results Section

Lines ~78-86 and Tables 3-10: Primary metric (frequency) is clearer, but dense reporting of p-values without full multiple-testing correction across tests (only within Kruskal-Wallis) risks inflation in exploratory context. Consider effect sizes (epsilon-squared mentioned but verify reporting) and suggest visualizations like heatmaps for nationality patterns across strokes/distances to enhance clarity.

Discussion and Conclusions

Lines ~40-224 (Discussion/Conclusions): Expands limitations (ix items, including multilevel modeling absence and ecological fallacy dismissal), but interpretations remain speculative—e.g., linking U.S. success to "pyramid" infrastructure (lines ~157-175) or genetics without direct measures. Ecological fallacy response (page 161) inadequately refutes group-level inference risks; strengthen by noting all analyses are aggregate. Tone down causal implications (e.g., "dominance" in conclusions) to purely descriptive patterns.

**********

7. PLOS authors have the option to publish the peer review history of their article (what does this mean?). If published, this will include your full peer review and any attached files.

If you choose “no”, your identity will remain anonymous but your review may still be made public.

**Do you want your identity to be public for this peer review?** For information about this choice, including consent withdrawal, please see our Privacy Policy.

Reviewer #3: **Yes:** Phornpot Chainok

Reviewer #4: No

**********

[NOTE: If reviewer comments were submitted as an attachment file, they will be attached to this email and accessible via the submission site. Please log into your account, locate the manuscript record, and check for the action link "View Attachments". If this link does not appear, there are no attachment files.]

To ensure your figures meet our technical requirements, please review our figure guidelines: https://journals.plos.org/plosone/s/figures

You may also use PLOS’s free figure tool, NAAS, to help you prepare publication quality figures: https://journals.plos.org/plosone/s/figures#loc-tools-for-figure-preparation.

NAAS will assess whether your figures meet our technical requirements by comparing each figure against our figure specifications.

# Author comment: Author response to Decision Letter 3

**DOI:** 10.1371/journal.pone.0352386.r006

19 May 2026

Responses to the Reviewer

Response: We thank the expert reviewer for the valuable and constructive evaluation. Below we address each comment point by point. All changes in the revised manuscript are marked.

Title Inconsistencies

Reviewer: The full title on page 1 ("Performance patterns and records in World Aquatics Masters Championships Which are the most frequently represented nations among the top-ten Masters swimmers?") mismatches the short title ("Origin of the fastest Masters Swimmers in the World"), which retains outdated "fastest" language despite revisions. Update the short title to align, e.g., "National Origins of Top Masters Swimmers," for consistency.

Response: We appreciate this important comment and apologize for the inconsistent titles. Accordingly, we have updated the short title to "National Origins of the Top-Ten Masters Swimmers". In addition, we have also revised the title as follows: “Performance Patterns and Records in the World Aquatics Masters Championships: Where Do the Most Frequently Represented Nations Among the Top-Ten Masters Swimmers Come From?”

Abstract & Introduction

Reviewer: Lines ~57-92 (abstract): Success is now explicitly defined, but the background still references "national origins of the most frequent Masters swimmers among the top-ten performers," which could be tightened to avoid implying causation. The introduction (lines ~94-126) appropriately hypothesizes U.S. dominance based on elite swimming but notes the demographic differences inadequately addressed in revisions.

Response: We thank the expert reviewer for his valuable observation. We revised parts of the abstract and introduction to ensure greater consistency between the title, hypothesis and results. In this context, we have now sought to avoid the term 'dominance' by placing greater emphasis on the demographic diversity of the most frequently top-ten finishers.

Methods Section

Reviewer: Lines ~127-202: Data recoding for Russian athletes (NIA to RUS) lacks a sensitivity analysis, as flagged by reviewers, potentially biasing recent rankings minimally but warranting verification. The unit of analysis (top-10 entries as independent) and lack of swimmer IDs for repeated measures are acknowledged, but no adjustment for participation rates or age-standardization is implemented, leaving selection bias and confounding risks. Add explicit data-cleaning protocols beyond flagging outliers (e.g., 1903s in Table 2).

Response: We thank the reviewer for raising this important point and would like to clarify the bounded nature of this recoding. The NIA code applied only to the 2023 and 2024 editions —2 of the 20 championship editions analyzed (10%). Our primary outcome is the cumulative frequency of top-ten appearances across 1986–2024, aggregated over 18 additional editions in which Russian athletes competed under the RUS code consistently. By construction, the recoding is ranking-preserving in the direction that matters: it restores continuity with 34 years of prior coding rather than introducing a novel classification. The marginal contribution of the 2023–2024 editions cannot exceed the number of Russian top-ten entries across those two editions for any given event, added to a base of 18 editions in which Russia's ranking position was already established. For events in which Russia was previously among the top five nations, the recoding reinforces a longstanding pre-existing representation rather than creating one; for events in which Russia was not historically among the top five, two editions alone cannot displace competing nations with multi-decadal representation. We have added a paragraph to the Methods section explicitly articulating this upper-bound argument and framing the recoding as historically continuity-preserving.

The absence of reliable unique swimmer identifiers in the World Aquatics archive is a known constraint of publicly archived Masters data and precludes the mixed-effects approach that would otherwise be preferred. Our rationale for proceeding with the swimmer-year entry as the unit of analysis is threefold: (i) the study's objective is to characterize national-level dominance, not individual career trajectories, and the relevant inferential unit for a national claim is a national event-year performance; (ii) inflation from repeated measures operates at the national level symmetrically—nations with prolific competitors contribute more entries, but this is precisely the "breadth and longevity of elite representation" construct we seek to measure; and (iii) the descriptive nature of the primary outcome (cumulative frequency) and the use of non-parametric Kruskal-Wallis tests minimize the impact of this assumption on distributional inference. We have expanded the limitations discussion to make these points explicit and we echo the reviewer's call for linked individual-level data in future work.

We argue that neither adjustment is appropriate for the specific construct our study targets. Participation rate adjustment would double-count the phenomenon we measure: a nation's participation in the World Masters Championships is not an extraneous confounder to be adjusted away—it is a constitutive component of "elite national representation". A nation that sends many swimmers of whom many reach the top-ten is by design more dominant in that event than a nation with a single representative, and this is what our cumulative frequency metric captures. Conditioning on participation rate would estimate a counterfactual orthogonal to our research question. Age-standardization would dilute elite-level signal: our sample is restricted to top-ten swimmer-year entries per event, stroke, and distance—the top-ten subset is already filtered on performance, and age distributions within this elite subset reflect the age at which nations actually produce top-ten performers (a substantively informative pattern, not a confounder). Age-standardization is the appropriate tool when comparing population-level prevalence across demographically different samples; it is not appropriate when the outcome is the joint distribution of national origin and competitive success within a pre-defined elite stratum. Data are already stratified by sex and five-year age group for all participation summaries, and the Kruskal-Wallis tests—being non-parametric—make no distributional assumptions that would require standardization. We have added a paragraph to Methods making these design choices explicit.

The data-cleaning pipeline was in fact more structured than the current manuscript conveys. We have added a dedicated Data Cleaning paragraph to Methods describing each step: (i) duplicate rows—concurrent matches on year, name, age group, stroke, and distance—were identified and removed; (ii) entries with missing, non-numeric, or zero race times were excluded from performance analyses but retained in participation counts when year and nationality were valid; (iii) race times exceeding three standard deviations above the event-specific mean were flagged for manual review, and in all but one instance were confirmed as physiologically plausible age-group performances and retained—the single exception, a 1903.00 s entry in the men's 50 m butterfly, was reviewed against the official archive, confirmed as a recording artifact, and retained for transparency (its exclusion does not alter any reported mean or statistical comparison, as noted in the Table 2 footnote); (iv) nationality codes were standardized against the IOC three-letter country-code list, with the recoding of "NIA" entries to "RUS" detailed in the preceding paragraph.

Results Section

Reviewer: Lines ~78-86 and Tables 3-10: Primary metric (frequency) is clearer, but dense reporting of p-values without full multiple-testing correction across tests (only within Kruskal-Wallis) risks inflation in exploratory context. Consider effect sizes (epsilon-squared mentioned but verify reporting) and suggest visualizations like heatmaps for nationality patterns across strokes/distances to enhance clarity.

Response: The reviewer correctly notes that our rationale for not applying a global correction across omnibus Kruskal-Wallis tests was asserted rather than argued; we now make the argument explicit. An additional correction across omnibus tests would be methodologically incongruent with the study's design for three reasons. First, the primary outcome is descriptive, not inferential: the central claim—the ranking of nations by cumulative frequency of top-ten appearances—does not depend on any p-value and is a direct tabulation. The Kruskal-Wallis tests provide secondary, contextualizing inference about whether mean race-time distributions differ across nations within each event. Second, Bonferroni is already applied at the pairwise level within each omnibus test, which is the adjustment most consequential for the specific between-nation claims we make (e.g., "Russian women were faster than others in the 50 m backstroke"). Third, across-test correction in exploratory descriptive studies is itself disputed in the methodological literature; Rothman (1990) and subsequent discussions have noted that universal cross-hypothesis correction can produce anti-conservative inference in the opposite direction (increased Type II error, suppression of substantively real findings) when applied to descriptive characterization of stratified data. We have clarified this reasoning in Methods and re-emphasized in Results that the substantive inference rests on reported effect sizes (epsilon-squared) and confidence intervals rather than on significance thresholds alone.

We confirm that epsilon-squared (ε²) is reported for every Kruskal-Wallis test in Tables 3–10 (see table footnotes). We have verified consistency across all eight tables and standardized the footnote language. No change to the reported values is required.

Also, we agree that a heatmap would make the nationality × event patterns substantially more readable than the eight-table tabulation. We have prepared a new two-panel figure (female and male) summarizing top-ten appearance frequency for each nation across all stroke × distance combinations. The figure is constructed from the aggregated data already reported in Tables 3–10, so no reanalysis of the raw archive is required. The figure is cited in Results and captioned in full; rows show the top five nations plus "Others" (all other countries pooled), columns show each stroke × distance event grouped by stroke, and cells report the cumulative count of top-10 appearances across the 20 championship editions.

Discussion & Conclusion

Reviewer: Lines ~40-224 (Discussion/Conclusions): Expands limitations (ix items, including multilevel modeling absence and ecological fallacy dismissal), but interpretations remain speculative—e.g., linking U.S. success to "pyramid" infrastructure (lines ~157-175) or genetics without direct measures. Ecological fallacy response (page 161) inadequately refutes group-level inference risks; strengthen by noting all analyses are aggregate. Tone down causal implications (e.g., "dominance" in conclusions) to purely descriptive patterns.

Response: We definetely agree with the expert reviewer that a multilevel model would be the methodologically preferred approach in principle, and we have expanded the Limitations section to acknowledge this explicitly. Two structural barriers prevent its implementation here. First, swimmer-level random effects require unique swimmer identifiers, which are not consistently retrievable from the public World Aquatics Masters archive across the 1986–2024 window; without stable IDs, the highest-resolution random-effect specification—performances within swimmers within nations—cannot be reliably implemented. Second, at the national/event/year level the primary outcome is intentionally an aggregate descriptor (cumulative top-ten frequency); modeling national-level variance as a random effect would shrink precisely the between-nation differences that constitute the research question, partially defeating the descriptive purpose. We have therefore retained the Kruskal-Wallis approach for secondary distributional comparisons within events, while making explicit in the Limitations that mixed-effects modeling—particularly performances nested within swimmers—represents a desirable extension contingent on access to linked individual-level data.

We thank the reviewer for prompting a more careful framing. The ecological fallacy arises when group-level associations are improperly transferred to individual-level claims. Our study is deliberately constructed to avoid this transfer: all inferential claims are made at the national level, matching the unit of analysis. We do not claim that an arbitrary swimmer from nation X is faster than an arbitrary swimmer from nation Y; we claim that nation X has produced more top-ten swimmer-year entries than nation Y across the period studied. The cumulative frequency metric is, by design, a count of national representations — a population-level descriptor of "elite breadth and longevity," not a parameter of individual ability. Where event-level distributional comparisons are reported, they describe the distribution of recorded race times for swimmers competing under each national flag in that event-year, not a latent national talent parameter. We have added a sentence to the Limitations explicitly cautioning readers against extending the national-level findings to individual-level interpretations.

In accordance with the reviewer's valuable comment, we have also replaced the term “dominance” with a more descriptive pattern throughout the conclusions.

Attachment Submitted filename: PONE-D-25-29285_Revision_2_Responses_auresp_3.docx

# Editor report: Decision Letter 3

**DOI:** 10.1371/journal.pone.0352386.r007

**Authors:** Rodrigo Zacca, Rodrigo Zacca, Rodrigo Zacca, Rodrigo Zacca

9 Jun 2026

Performance Patterns and Records in the World Aquatics Masters Championships: Where Do the Most Frequently Represented Nations Among the Top-Ten Masters Swimmers Come From?

PONE-D-25-29285R3

Dear Dr. Knechtle,

We’re pleased to inform you that your manuscript has been judged scientifically suitable for publication and will be formally accepted for publication once it meets all outstanding technical requirements.

Within one week, you’ll receive an e-mail detailing the required amendments. When these have been addressed, you’ll receive a formal acceptance letter and your manuscript will be scheduled for publication.

An invoice will be generated when your article is formally accepted. Please note, if your institution has a publishing partnership with PLOS and your article meets the relevant criteria, all or part of your publication costs will be covered. Please make sure your user information is up-to-date by logging into Editorial Manager at Editorial Manager® and clicking the ‘Update My Information' link at the top of the page. For questions related to billing, please contact billing support.

If your institution or institutions have a press office, please notify them about your upcoming paper to help maximize its impact. If they’ll be preparing press materials, please inform our press team as soon as possible -- no later than 48 hours after receiving the formal acceptance. Your manuscript will remain under strict press embargo until 2 pm Eastern Time on the date of publication. For more information, please contact onepress@plos.org.

Kind regards,

Rodrigo Zacca, Ph.D

Academic Editor

PLOS One

Additional Editor Comments (optional):

Dear Dr. Beat Knechtle,

The revised manuscript is satisfactory and meets the journal’s standards for publication.

I would like to thank the authors for their patience and careful revisions, as well as the reviewers for their expertise, effort, and availability.

I warmly congratulate the authors on the quality of the work and recommend acceptance for publication in PLOS ONE.

Sincerely,

Academic Editor

Reviewers' comments:

# Editor report: Acceptance letter

**DOI:** 10.1371/journal.pone.0352386.r008

**Authors:** Rodrigo Zacca, Rodrigo Zacca, Rodrigo Zacca, Rodrigo Zacca

PONE-D-25-29285R3

PLOS One

Dear Dr. Knechtle,

I'm pleased to inform you that your manuscript has been deemed suitable for publication in PLOS One. Congratulations! Your manuscript is now being handed over to our production team.

At this stage, our production department will prepare your paper for publication. This includes ensuring the following:

* All references, tables, and figures are properly cited

* All relevant supporting information is included in the manuscript submission,

* There are no issues that prevent the paper from being properly typeset

You will receive further instructions from the production team, including instructions on how to review your proof when it is ready. Please keep in mind that we are working through a large volume of accepted articles, so please give us a few days to review your paper and let you know the next and final steps.

Lastly, if your institution or institutions have a press office, please let them know about your upcoming paper now to help maximize its impact. If they'll be preparing press materials, please inform our press team within the next 48 hours. Your manuscript will remain under strict press embargo until 2 pm Eastern Time on the date of publication. For more information, please contact onepress@plos.org.

You will receive an invoice from PLOS for your publication fee after your manuscript has reached the completed accept phase. If you receive an email requesting payment before acceptance or for any other service, this may be a phishing scheme. Learn how to identify phishing emails and protect your accounts at https://explore.plos.org/phishing.

If we can help with anything else, please email us at customercare@plos.org.

Thank you for submitting your work to PLOS ONE and supporting open access.

Kind regards,

PLOS ONE Editorial Office Staff

on behalf of

Dr. Rodrigo Zacca

Academic Editor

PLOS One
